# Vitamin B_12_ is a limiting factor for induced cellular plasticity and tissue repair

**DOI:** 10.1038/s42255-023-00916-6

**Published:** 2023-11-16

**Authors:** Marta Kovatcheva, Elena Melendez, Dafni Chondronasiou, Federico Pietrocola, Raquel Bernad, Adrià Caballe, Alexandra Junza, Jordi Capellades, Adrián Holguín-Horcajo, Neus Prats, Sylvere Durand, Meritxell Rovira, Oscar Yanes, Camille Stephan-Otto Attolini, Guido Kroemer, Manuel Serrano

**Affiliations:** 1 Institute for Research in Biomedicine (IRB Barcelona), Barcelona Institute of Science and Technology (BIST), Barcelona, Spain; 2 Department of Biosciences and Nutrition, Karolinska Institutet, Huddinge, Sweden; 3 Universitat Rovira i Virgili, Department of Electronic Engineering, IISPV, Tarragona, Spain; 4 CIBER de Diabetes y Enfermedades Metabólicas Asociadas (CIBERDEM), Instituto de Salud Carlos III, Madrid, Spain; 5 Institut d’Investigació Sanitària Pere Virgili (IISPV), Metabolomics Platform, Reus, Spain; 6 Department of Physiological Science, School of Medicine, Universitat de Barcelona (UB), L’Hospitalet de Llobregat, Spain; 7 Pancreas Regeneration: Pancreatic Progenitors and Their Niche Group, Regenerative Medicine Program, Institut d’Investigació Biomèdica de Bellvitge (IDIBELL), L’Hospitalet de Llobregat, Spain; 8 Metabolomics and Cell Biology Platforms UMS AMMICa/UMR 1138, Institut Gustave Roussy, Villejuif, France; 9 Equipe labellisée par la Ligue contre le cancer, Centre de Recherche des Cordeliers, Inserm U1138, Université de Paris, Sorbonne Université, Institut Universitaire de France, Paris, France; 10 Institut du Cancer Paris CARPEM, Department of Biology, Hôpital Européen Georges Pompidou, AP-HP, Paris, France; 11 Catalan Institution for Research and Advanced Studies (ICREA), Barcelona, Spain; 12 Altos Labs, Cambridge Institute of Science, Cambridge, UK

**Keywords:** Reprogramming, Cell biology, Metabolism, Regeneration

## Abstract

Transient reprogramming by the expression of OCT4, SOX2, KLF4 and MYC (OSKM) is a therapeutic strategy for tissue regeneration and rejuvenation, but little is known about its metabolic requirements. Here we show that OSKM reprogramming in mice causes a global depletion of vitamin B_12_ and molecular hallmarks of methionine starvation. Supplementation with vitamin B_12_ increases the efficiency of reprogramming both in mice and in cultured cells, the latter indicating a cell-intrinsic effect. We show that the epigenetic mark H3K36me3, which prevents illegitimate initiation of transcription outside promoters (cryptic transcription), is sensitive to vitamin B_12_ levels, providing evidence for a link between B_12_ levels, H3K36 methylation, transcriptional fidelity and efficient reprogramming. Vitamin B_12_ supplementation also accelerates tissue repair in a model of ulcerative colitis. We conclude that vitamin B_12_, through its key role in one-carbon metabolism and epigenetic dynamics, improves the efficiency of in vivo reprogramming and tissue repair.

## Abstract (web summary)

Kovatcheva et al. show that vitamin B_12_ improves the efficiency of in vivo reprogramming and tissue repair through its functions in one-carbon metabolism and epigenetic modulation.

## Main

Cellular reprogramming consists of the loss of differentiated cell identity followed by the acquisition of embryonic stem pluripotency, which can be achieved by the simultaneous expression of the transcription factors OCT4, SOX2, KLF4 and MYC (OSKM; in mice encoded by *Pou5f1*, *Sox2*, *Klf4* and *Myc*, respectively)^1^. During recent years, it has become evident that this process involves intermediate states in which cells acquire various degrees of plasticity and differentiation potential, which may have broad implications in regenerative medicine and organ repair (reviewed in ref. ^2^). Continuous expression of OSKM in mice can recapitulate full reprogramming to pluripotency, a process that culminates with the generation of teratomas^3^. Interestingly, transient expression of OSKM leads to molecular and physiological features of rejuvenation, including an enhanced capacity for tissue regeneration^4–9^. Nevertheless, in vivo reprogramming via OSKM remains a poorly understood process, with low efficiency and high risks, including teratoma and cancer development^3,10,11^. Thus, we sought to unravel new molecular mechanisms of in vivo reprogramming that could be harnessed to manipulate cell plasticity and tissue repair.

Given the unique metabolic requirements of in vitro reprogramming^12,13^, we hypothesized that unique metabolic requirements may also operate during in vivo reprogramming. As a new approach, we considered the gut microbiota as a commensal community in metabolic equilibrium with its host. Indeed, the microbiota is sensitive to perturbations in host physiology, capable of adapting and rewiring itself based on nutrient availability and depletion^14^, a process known as the host–gut microbiota metabolic interaction^15^. We reasoned that analysis and manipulation of the microbiota could provide new insights into the metabolic requirements of in vivo reprogramming.

## In vivo reprogramming is dependent on the microbiota

**Fig. 1:**
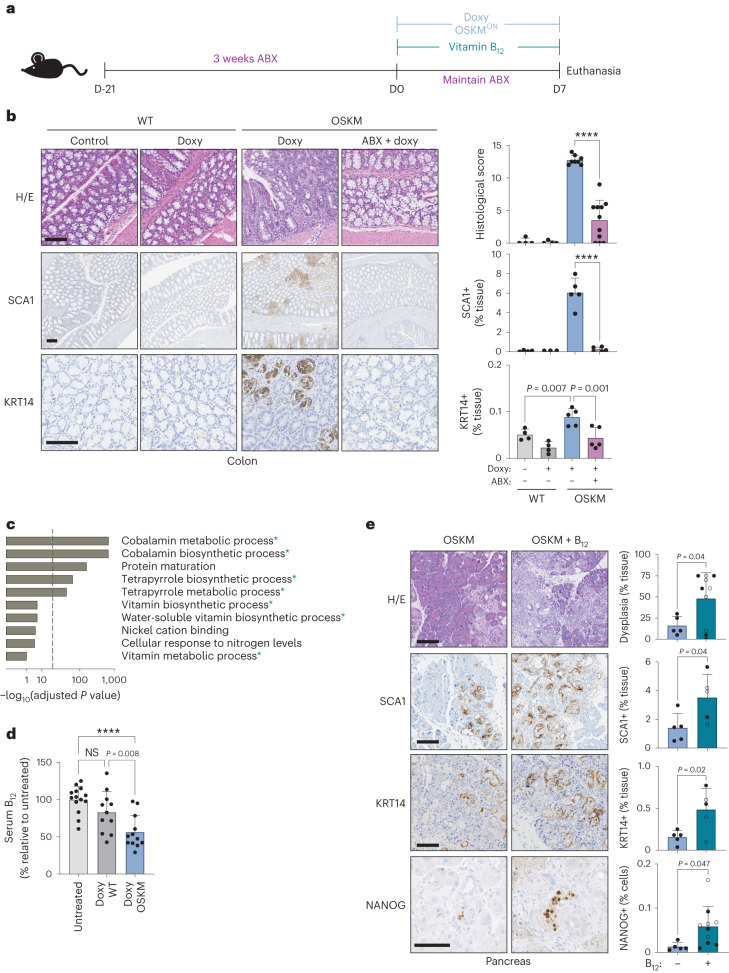
In vivo OSKM reprogramming requires the gut microbiota and is enhanced by vitamin B_12_ supplementation. **a**, Mice were pretreated with an antibiotic cocktail administered in the drinking water for 3 weeks (ABX) before, and during, 7 d of doxycycline administration (doxy), with or without vitamin B_12_ supplementation according to the schematic. **b**, Representative histology images and quantification of a blinded histological score, SCA1 staining and KRT14 staining. *n* = 4 mice (WT; 3 M 1 F), *n* = 8 (OSKM + doxy; 4 M 4 F), *n* = 11 (OSKM + doxy + ABX 4 M 7 F); a representative subset of animals was analysed for SCA1 and KRT14. Scale bar, 100 µm. **c**, GO pathway analysis of differentially abundant microbial gene signatures in the metagenome sequencing of stool samples. Changes in microbial gene abundance between day 7 and day 0 were compared in a subset of WT (*n* = 4; 2 M 2 F) and OSKM (*n* = 4; 2 M 2 F) mice from **b**. See Supplementary Table 1 for complete gene list. The overlap between GO terms and the 200 most differentially depleted or enriched genes was scored using standard hypergeometric tests and GO terms above a threshold of 30% FDR are shown (for all GO terms, see Supplementary Table 2). Processes marked with an asterisk are directly related to cobalamin metabolism. Dashed line indicates 5% FDR cut-off. **d**, Serum holoTC (biologically available vitamin B_12_) levels as measured by ADVIA immunoassay in untreated mice or WT and OSKM mice treated with doxycycline for 7 d. *n* = 14 mice (untreated; 6 M 8 F), *n* = 11 (doxy WT doxy; 7 M 4 F), *n* = 12 (doxy OSKM doxy; 6 M 6 F). **e**, OSKM mice received vitamin B_12_ supplementation co-administered with doxycycline as indicated and representative images and quantification are shown for the indicated markers in the pancreas. Mice marked by an open circle received both B_12_ and folate (B_9_) supplementation (not significant (NS) difference for B_12_ versus B_12_ + folate; see text for details). *n* = 5 mice (OSKM; 2 M 3 F), *n* = 10 mice (OSKM + B_12_; 4 M 6 F); a representative subset of *n* = 5 animals per group was analysed for SCA1 and KRT14. Scale bars, 100 µm. Bar graphs represent the average ± s.d.; ****P < 0.0001 by two-tailed Student’s *t*-test. Source data

**Extended Data Fig. 1:**
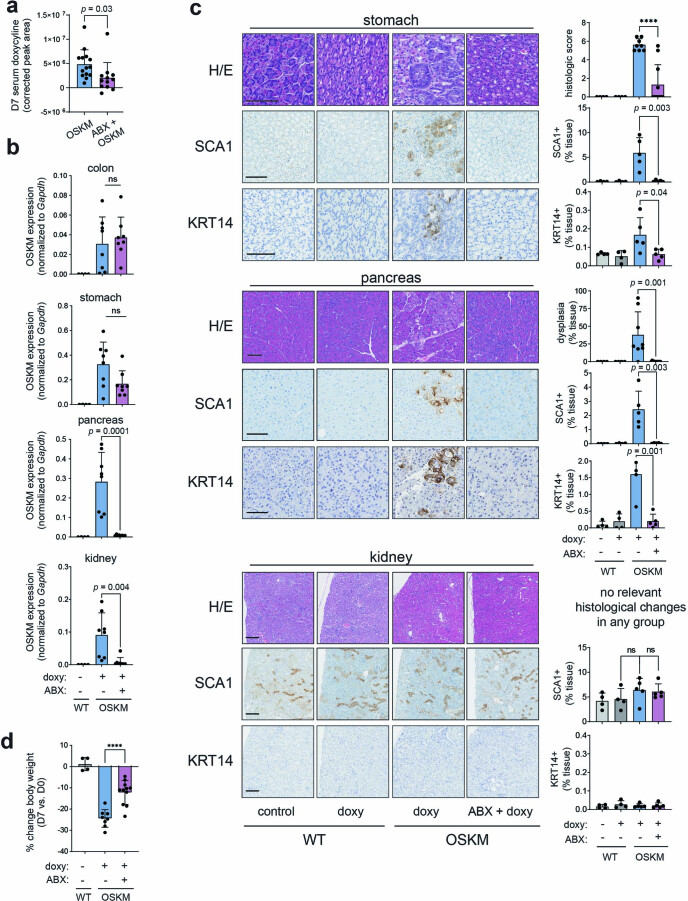
Broad-spectrum antibiotic treatment inhibits in vivo reprogramming. (a-d) Mice were treated with doxycycline (doxy) and antibiotics (ABX) as described in Fig. 1a. (a) Serum was harvested from OSKM mice after 7 days of doxycycline treatment (OSKM n = 14; 5 M 9 F), or after ABX + doxycycline treatment (ABX + OSKM n = 12; 3 M 9 F) and doxycycline levels were analyzed by mass spectrometry. The total area of the doxycycline peak corrected for background levels for each mouse is shown. (b-d) Representative subsets of mice from Fig. 1b n = 4 mice (WT; 3 M 1 F), n = 8 (OSKM + doxy; 4 M 4 F), n = 11 (OSKM + doxy + ABX 4 M 7 F) were used for all analyses (b) Tissues were harvested on day 7 post doxycycline initiation and the expression of the OSKM transgene was assessed by RT-qPCR. (c) Representative histology images of the indicated treatments and markers are shown (left) and quantified (right). Histologic score for stomach dysplasia and inflammation was assigned by a blinded pathologist. No relevant morphologic changes were observed in the kidney cortex and medulla in any of the mice. (d) Body weights of mice from the indicated treatment groups, expressed as a percent change on day 7 post-doxycycline as compared to pre-doxycycline (day 0). Scale bars are 100 µm. Graphs represent average ± SD; ****p < 0.0001 by two-tailed Student’s t-test.

To study modulators of in vivo reprogramming, we used a previously described mouse model in which doxycycline drives systemic, inducible OSKM expression^3,9,16,17^. On a short timescale (7 days), OSKM induction causes focal regions of abnormal tissue architecture, correlating with the appearance of rare NANOG-positive cells (a marker of embryonic pluripotency) predominantly in the pancreas, colon and stomach^3^. We first asked whether the microbiota was important for in vivo reprogramming by disrupting it with a commonly used, broad-spectrum cocktail of antibiotics (ABX): ampicillin, metronidazole, neomycin and vancomycin^18^. We administered ABX for 3 weeks before and during the 7 days of OSKM induction (Fig. 1a). We noted that mice treated with ABX had very low levels of serum doxycycline (Extended Data Fig. 1a), therefore precluding the induction of OSKM in organs beyond the gastrointestinal tract (Extended Data Fig. 1b). Nevertheless, doxycycline efficiently induced OSKM in the colon and stomach in the presence of ABX (Extended Data Fig. 1b). Strikingly, despite strong transgene induction, reprogramming was significantly reduced in the colon and stomach of ABX-treated mice (Fig. 1b and Extended Data Fig. 1c). Reduction in reprogramming was also reflected in the reduced abundance of SCA1-positive and KRT14-positive cells (Fig. 1b and Extended Data Fig. 1c), markers of early and advanced stages of intermediate in vivo reprogramming, respectively^19^. Consistent with low levels of reprogramming, ABX-treated mice lost significantly less weight than mice with normal levels of reprogramming (Extended Data Fig. 1d). These results indicate that the microbiota is critical for the successful reprogramming of tissues in vivo.

## In vivo reprogramming causes microbial dysbiosis

**Extended Data Fig. 2:**
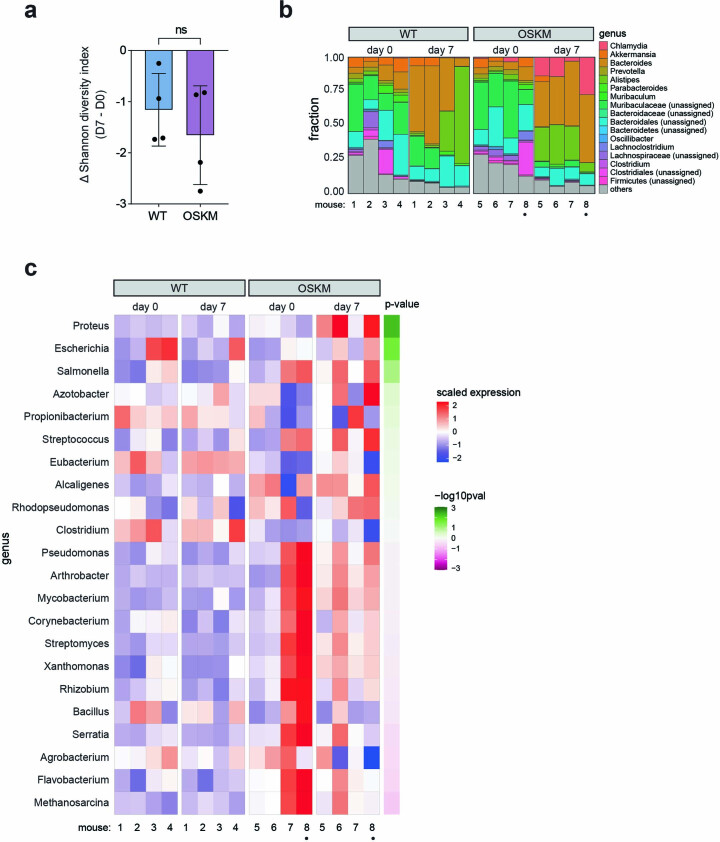
Metagenomic analysis of fecal bacteria during in vivo reprogramming. (a-c) Mice were treated with doxycycline for 7 days as described in Fig. 1a. Stool from subset of WT (n = 4; 2 M 2 F) and OSKM (n = 4 2 M 2 F) mice was analyzed before (pre; day 0) and after seven days (post; day 7) of doxycycline (stool was collected repeatedly from the same mice). (a) The change in the Shannon diversity index on day 7 as compared to day 0 in the indicated treatment groups. Negative values indicate reduced diversity post-doxycyline. Graph represents average ± SD; ns p˃0.05 by two-tailed Student’s t-test. (b) Microbial stool diversity at the genus level, as categorized by 16 S rRNA sequencing. The 17 genera with the highest abundance are shown. “Unassigned” refers to bacterial species that could not be assigned a specific genus. “Others” includes assigned bacteria with low counts, as well as archaea. See Supplementary Table 1 for complete list. Mouse 8, marked with a dot (●), had a large proportion of host (murine) DNA contamination in the stool sample. (c) Normalized abundance of genera capable of de novo synthesis of vitamin B12 in the indicated conditions. Genera are ordered by significance in the day7/day0 (OSKM) vs day7/day0 (WT) interaction, and raw p-values (by two-tailed Wald test (DESeq2)) for this score are shown. Source data

Given the profound impact that disruption of the microbiota had on in vivo reprogramming, we reasoned that a functional analysis of microbial changes during this process could illuminate previously unknown requirements for reprogramming. To this end, we isolated bacterial DNA from paired stool samples of both OSKM-expressing mice and wild-type (WT) littermate control mice before and after 7 days of doxycycline treatment, and performed shotgun metagenome sequencing^20^ (Extended Data Fig. 2a–c and Supplementary Tables 1 and 2). In both WT and OSKM mice, the microbial diversity as measured by the Shannon index decreased following 7 days of doxycycline treatment, with the most profound loss of diversity occurring in reprogrammed mice (Extended Data Fig. 2a). At a genus level, reprogrammed mice were characterized by a relative expansion of *Chlamydia*, *Bacteriodes* and *Alistipes* spp. and a relative contraction of *Muribaculaceae* spp. (Extended Data Fig. 2b). *Muribaculaceae* have been reported to contract during inflammatory colonic injury^21^, which shares features with in vivo reprogramming including inflammation and loss of differentiated cell identity^22^. *Alistipes* on the other hand, have been reported to promote colonic interleukin (IL)-6 production^23^, which is an important mediator of in vivo reprogramming^16^.

## In vivo reprogramming reduces systemic vitamin B_12_ levels

Our whole-genome approach allowed us to investigate changes not only in bacterial species abundance, but also in gene composition and ontology groups, which could uncover pathways relevant to reprogramming. Remarkably, we found that microbial gene modules related to the biosynthesis and metabolism of cobalamin (vitamin B_12_) dominated the bacterial Gene Ontology (GO) groups altered during reprogramming (Fig. 1c and Supplementary Table 2). Under conditions of disrupted cobalamin bioavailability, competition for vitamins can shift the relative abundance of cobalamin-producing and cobalamin-utilizing bacteria in a process referred to as ‘corrinoid remodelling’^14,24^. We found microbial changes consistent with this phenomenon in reprogramming: the few genera of bacteria able to synthesize B_12_ (~20 genera)^25^ were generally enriched in OSKM mice after 7 days of doxycycline, with *Proteus*, *Escherichia* and *Salmonella* being most significantly enriched among the B_12_ synthesizers (Extended Data Fig. 2c and Supplementary Table 2).

**Extended Data Fig. 3:**
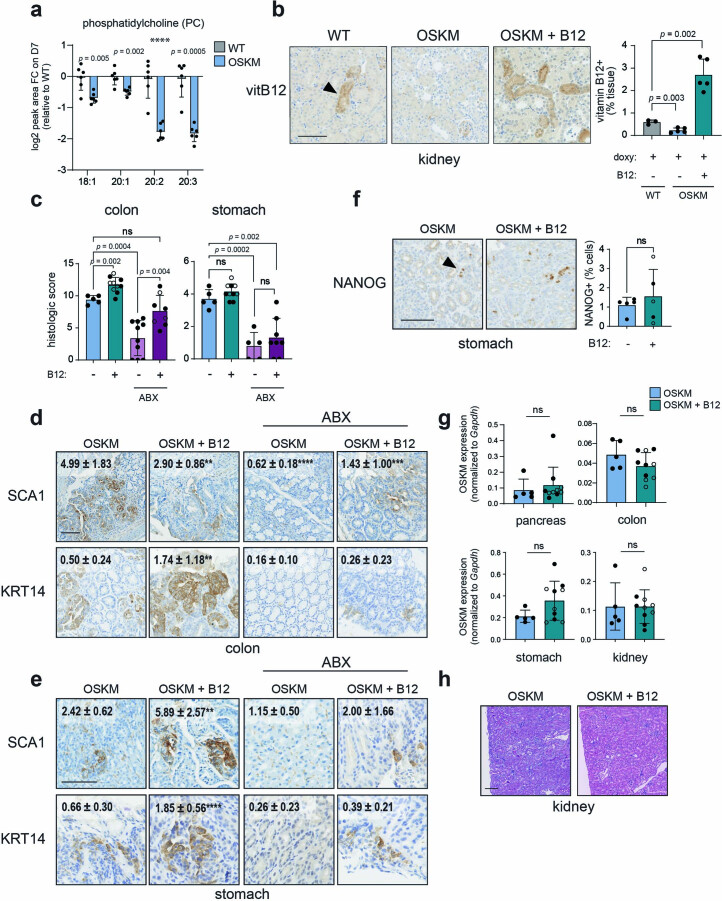
Vitamin B12 deficiency limits in vivo reprogramming. (a) Abundance of phosphatidylcholine (PC) species in the serum during reprogramming. WT and OSKM mice (n = 6 per group; 3 M 3 F) were treated with doxycycline for 7 days and paired serum samples were taken pre- (D0) and post (D7) doxycyline for metabolic analysis by mass spectrometry. The relative abundance of the indicated PC moieties are given as a log2 fold change (FC) as compared to WT on D7. P-values represent the comparison between WT and OSKM groups by two-tailed Student’s t-test for each PC moiety. (b) Vitamin B12 immunohistochemistry in the kidney. WT and OSKM mice were treated with doxycycline with or without vitamin B12 as indicated for 7 days. Representative images of kidneys stained for vitamin B12 are shown (left) and total staining is quantified (right). WT doxy (n = 3; 2 M 1 F); OSKM doxy (n = 5; 5 M) and OSKM doxy + B12 (n = 5; 1 M 4 F). (c-h) Analysis of reprogramming in mice treated as described in Fig. 1a, with treatments as indicated. (c) Histologic score of reprogramming was assigned by a blinded histopathologist in the colon (left) and stomach (right). n = 5 mice (OSKM 2 M 3 F and OSKM + ABX, stomach 5 F), n = 10 (OSKM + B12 4 M 6 F and OSKM + ABX, colon 10 F), n = 8 (OSKM + ABX + B12 3 M 5 F). (d-e) Immunohistochemistry of SCA1 and KRT14 in the colon (d) and stomach (e). Representative images and quantification (average ± SD; inset) from a subset of n = 5 mice per condition are shown. Asterisks represent comparison to the OSKM condition by one-way ANOVA; **p < 0.01, ***p < 0.001, ****p < 0.0001. (f-h) Analysis of mice from Fig. 1e treated with doxycycline, with or without vitamin B12 supplementation as indicated. (f) NANOG immunohistochemistry in the stomach of n = 5 representative mice per group. (g) Expression of the OSKM transgene assessed by RT-qPCR in the indicated tissues on D7 post doxy. n = 5 mice (OSKM; 2 M 3 F), n = 10 mice (OSKM + B12; 4 M 6 F). (h) Representative images of H/E staining of the kidney. No relevant histologic changes were observed in the kidney of any of the mice. Scale bars are 100 µm. Mice marked by an open circle received both B12 and folate supplementation (n.s. difference for B12 vs B12 + folate; see text for details). Graphs represent average ± SD; p-values are by two-tailed Student’s t-test unless otherwise indicated. Source data

The observed changes in the gut microbiota could be indicative of a systemic deficit in B_12_, affecting not only the microbiota but also the entire physiology of the host. To test this, we examined systemic vitamin B_12_ levels in the serum during reprogramming, which were significantly reduced in OSKM mice after 7 days of doxycycline administration (Fig. 1d). The liver is one of the organs with the greatest demand for vitamin B_12_ (ref. ^26^) and, as such, is sensitive to B_12_ deficiency^27^. In rodents, this manifests as depletion of phosphatidylcholines (PCs)^28^, which are produced in large quantities by the liver in a B_12_-dependent manner. We saw that PCs were significantly reduced in the serum of reprogrammed mice as compared to WT mice treated with doxycycline (Extended Data Fig. 3a). Importantly, the liver of OSKM mice does not exhibit histological changes after 7 days of doxycycline^9^, making the reduction in PCs unlikely to reflect liver dysfunction as a result of reprogramming. The kidney is another organ that is refractory to reprogramming in our mouse model^3^; however, we did observe a significant depletion of vitamin B_12_ from the proximal tubules during reprogramming (Extended Data Fig. 3b). The kidney is the primary site of B_12_ concentration and storage in rodents, from where it is released for use by other organs upon systemic deficiency^27^^,29–31^. Collectively, these results suggest that vitamin B_12_ becomes systemically depleted during in vivo reprogramming, affecting both the colonic microbiota and the host.

## Vitamin B_12_ supplementation improves in vivo reprogramming

Given the systemic reduction of vitamin B_12_ during in vivo reprogramming, we hypothesized that B_12_ supplementation could enhance reprogramming under normal conditions (that is, in the absence of ABX). Indeed, vitamin B_12_ supplementation significantly improved in vivo reprogramming in the pancreas, colon and stomach, as evaluated by the extent of histological dysplasia and SCA1 or KRT14 levels (Fig. 1e and Extended Data Fig. 3c–e). B_12_ also increased the number of NANOG^+^ cells, a marker of full pluripotency, in the pancreas (Fig. 1e and Extended Data Fig. 3f). B_12_ administration did not affect transgene expression (Extended Data Fig. 3g). Even after B_12_ supplementation, we could not detect histological evidence of reprogramming in the kidney (Extended Data Fig. 3h). However, we did observe a significant increase of vitamin B_12_ stores within the kidney after supplementation (Extended Data Fig. 3b), indicating that B_12_ absorption, distribution and storage were occurring normally in the reprogrammed mice.

We also wondered if B_12_ supplementation could rescue the reprogramming defect of ABX-treated mice. Interestingly, B_12_ supplementation was able to partially rescue reprogramming in the colon (Extended Data Fig. 3c–e). This supports the concept that an important role of the microbiota during murine reprogramming is to increase the dietary supply of B_12_ through coprophagy. Another B vitamin that is partly supplied by the microbiota in rodents and humans is vitamin B_9_ (folate)^32^, which is functionally related to B_12_ (ref. ^33^). However, co-supplementation of B_12_ and B_9_ was indistinguishable from B_12_ alone (Fig. 1e and Extended Data Fig. 3c,f,g). Collectively, these results demonstrate that vitamin B_12_ is a limiting factor for in vivo reprogramming.

## One-carbon metabolism drives vitamin B_12_ demand during reprogramming

**Fig. 2:**
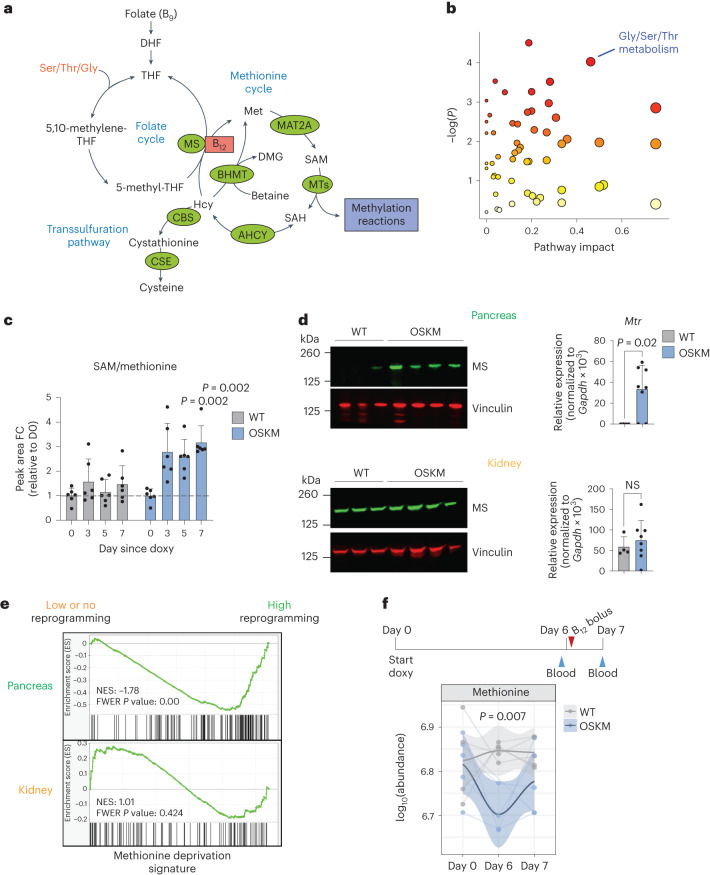
Tissues undergoing in vivo reprogramming exhibit an increased demand of 1C metabolism. **a**, Summary of the mammalian folate and methionine cycles (1C metabolism) and the transsulfuration pathway. Enzymes are marked in green. Coenzyme vitamin B_12_ is marked in red. DHF, dihydrofolate; THF, tetrahydrofolate; MTs, methyltransferases; ACHY, adenosylhomocysteinase; CSE, cystathionine gamma-lyase; DMG, dimethylglycine; Ser, serine; Thr, threonine; Gly, glycine. Figure adapted from ref. ^33^, Springer Nature Limited. **b**, Changes in metabolic pathways during reprogramming. MetaboAnalyst (4.0)^91^ was used to assess the annotated metabolites identified in the serum of paired OSKM mice (*n* = 6; 3 M 3 F) at day 5 versus day 0 of doxycycline treatment (serum was collected repeatedly from the same mice). Colour gradient from white to red indicates the *P* value; red is most significant. Gly/Ser/Thr metabolism (KEGG map00260) is highlighted. See Supplementary Table 3 for all metabolites, pathways and scores. **c**, Fold change (FC) of SAM/Met ratio detected by mass spectrometry from **b** on the indicated days. *P* values represent significant difference between OSKM and WT mice. **d**, Levels of MS (encoded by *Mtr*) by immunoblot and RT–qPCR in the pancreas (upper) and kidney (lower) from WT (*n* = 4; 3 M 1 F) and OSKM (*n* = 8; 4 M 4 F) mice treated with doxycycline for 7 d. Representative mice are shown in the immunoblot. **e**, Previously published RNA-seq data^16^ from the pancreas (highly prone to reprogramming; green) and kidney (refractory to reprogramming; orange) of OSKM-*Cdk2na*^−*/*−^(low or absent reprogramming) and OSKM-*Tp53*^−/−^ (high reprogramming) mice were used to perform GSEA against a published signature (MsigDB: M13537) of Met deprivation^39^. **f**, WT and OSKM mice (*n* = 5 per group; 5 M) were treated with doxycycline and a bolus of vitamin B_12_ as shown in the schematic. Met levels were measured in the indicated serum samples by mass spectrometry. Only *n* = 4 WT (day 0) and OSKM (day 6) mice are represented, as the blood volume was insufficient. Welch’s two-sample *t*-test was used to evaluate differences between groups on day 6. Bar graphs represent the average ± s.d.; *P* values determined by a two-tailed Student’s *t*-test. Source data

**Extended Data Fig. 4:**
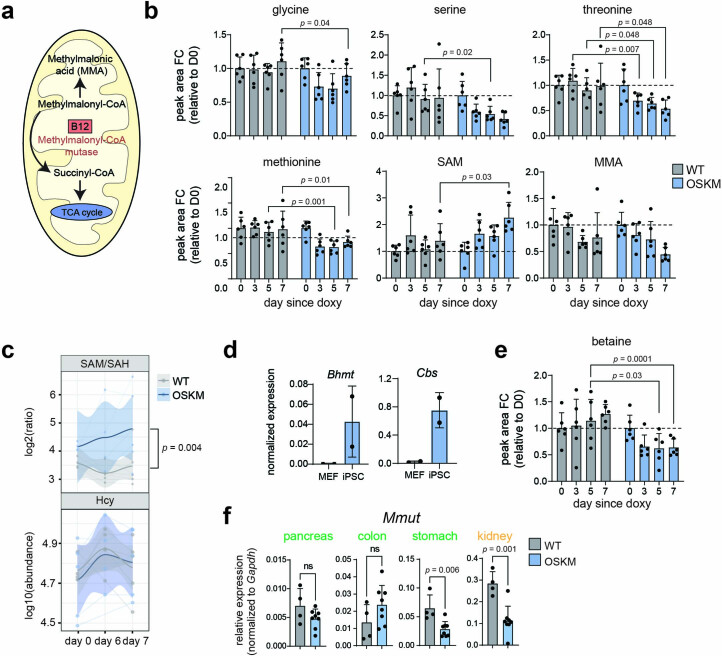
Vitamin B12 and 1-carbon (1 C) metabolism markers during in vivo reprogramming. (a) Schematic of methylmalonyl-coA mutase (MUT) enzymatic activity in the mitochondria, Figure adapted from ref. ^26^, Springer Nature Limited. TCA: tricarboxylic acid. (b) Metabolites of the Gly/Ser/Thr metabolism pathway (KEGG map00260) identified in the serum of WT and OSKM mice (n = 6 mice per genotype; 3 M 3 F) during a time course of doxycycline administration. Fold change (FC) of the peak area of the indicated metabolites as detected by mass spectrometry is shown relative to (D0) for the respective groups (that is WT and OSKM). P-values represent significant difference between OSKM and WT mice on the indicated day. Serum was collected repeatedly from the same mice. (c) WT and OSKM mice (n = 5 per group; 5 M) were treated with doxycycline and given a bolus of vitamin B12 on day 6, as schematized in Fig. 2f. Serum (repeated measurements) was analyzed by mass spectrometry prior to doxycycline (day 0), prior to the B12 bolus (day 6), and 24 hours after the bolus (day 7). The ratio of SAM/SAH abundance (upper) and total abundance of homocysteine (Hcy, lower) are shown. P-value represents comparison of WT vs. OSKM by two-way ANOVA. (d) Expression of Bhmt and Cbs in previously published RNA sequencing data from n = 2 paired parental MEFs and induced pluripotent stem cells (iPSC), generated by doxycycline-induced OSKM expression. Data source: 40. (e) Serum analysis of betaine in the mice described in panel (b). (f) Expression of methylmalonyl-coA mutase (Mmut) in WT (n = 4) and OSKM (n = 8) mice from Fig. 1b. Mice were treated with doxycycline for 7 days and RT-qPCR was performed in tissues that do (pancreas, stomach, colon; green) and do not (kidney; orange) exhibit histologic signs of reprogramming. Bar graphs represent average ± SD; p-values by two-tailed Student’s t-test unless otherwise indicated. Source data

In both humans and mice, vitamin B_12_ is used as a cofactor by only two enzymes: methionine synthase (MS) and methylmalonyl-CoA mutase (MUT)^26^. MS uses B_12_ as a cofactor to regenerate methionine (Met) from homocysteine (Hcy), forming an integral part of one-carbon (1C) metabolism (Fig. 2a). Met is used to synthesize *S*-adenosylmethionine (SAM), the universal methyl donor for all methylation reactions^33^. The nuclear-encoded mitochondrial enzyme MUT uses B_12_ as a cofactor for the catabolism of branched-chain amino acids via isomerization of methylmalonyl-CoA to succinyl-CoA, for use in the tricarboxylic acid cycle (Extended Data Fig. 4a)^26^.

As a first approach to investigate B_12_-dependent metabolism during in vivo reprogramming, we performed untargeted serum metabolomics. The metabolic pathway with the strongest (by pathway impact) and most significant changes during reprogramming was ‘glycine (Gly), serine (Ser), threonine (Thr) metabolism’ (Kyoto Encyclopedia of Genes and Genomes (KEGG) pathway map00260; Fig. 2b and Supplementary Table 3). These three amino acids, together with Met, feed 1C metabolism (Fig. 2a). Notably, Thr is known to be critical for the generation of SAM during in vitro reprogramming and in the maintenance of pluripotent cells^12,34,35^. In OSKM as compared to WT mice, we saw significant depletion of all four of these amino acids (Gly, Ser, Thr and Met), concomitant with an increase in SAM and an increase in the SAM/Met ratio, progressively over the course of reprogramming (Fig. 2c and Extended Data Fig. 4). The increased SAM/Met ratio was indicative of methylation cycle activation, which occurs during in vitro reprogramming^13^ and in cultured pluripotent cells^12,34,35^. Neither Hcy nor *S*-adenosyl-l-homocysteine (SAH), two important 1C metabolites, were detected via untargeted metabolomics, so we performed a separate, targeted serum metabolomic analysis. We found that the SAM/SAH ratio, known as the ‘methylation index’ because it indicates the methylation capacity of an organism^36^, was significantly increased during in vivo reprogramming (Extended Data Fig. 4c). We did not observe changes in Hcy (Extended Data Fig. 4c). Although Hcy accumulation can occur clinically as a result of B_12_ insufficiency in humans^26^, we observed that induced pluripotent stem (iPS) cells upregulate the expression of genes encoding two main consumers of Hcy: cystathionine beta-synthase (CBS, which initiates the transsulfuration pathway) and betaine-homocysteine methyltransferase (BHMT, which synthesizes Met using Hcy and betaine in an MS-independent manner; Fig. 2a and Extended Data Fig. 4d). In support of this, we found that serum betaine levels were significantly decreased during in vivo reprogramming (Extended Data Fig. 4e), which may serve as an additional or alternate source of Met generation.

On the other hand, methylmalonic acid (MMA), the substrate of MUT, showed no significant differences between OSKM mice and WT littermate controls treated with doxycycline (Extended Data Fig. 4b). The MMA levels, along with decreases in *Mmut* expression in several organs (Extended Data Fig. 4f), suggested that MUT’s enzymatic activity does not become limiting during reprogramming. It is important to note that while serum accumulation of MMA and Hcy are sensitive biomarkers of vitamin B_12_ deficiency in humans^26^, this is not the case in mice^37,38^.

**Extended Data Fig. 5:**
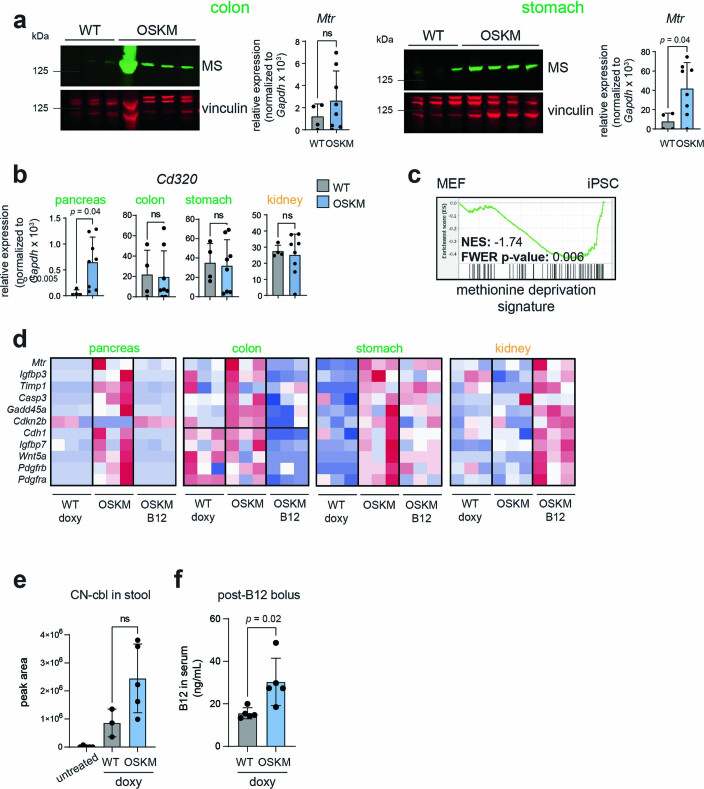
Reprogramming induces a methionine deprivation signature that is relieved by vitamin B12. (a) Immunoblot (left) and RT-qPCR (right) to measure levels of methionine synthase (encoded by Mtr) in the colon and stomach of WT (n = 4, 3 M 1 F) and OSKM (n = 8, 4 M 4 F) mice from Fig. 1b treated with doxycycline for 7 days. Representative mice are shown in the immunoblot. (b) Expression of Cd320 measured by RT-qPCR in the indicated tissues from WT and OSKM mice described in panel (a). (c) Previously published RNAseq data of iPS cells and their parental MEFs (data source: 40) was used to perform gene set enrichment analysis against a published signature of methionine deprivation (msigdb: M13537; ref. ^39^). (d) RT-qPCR analysis in the indicated tissues from a subset of mice from Fig. 1. The genes were derived from the methionine deprivation signature shown in (c); see Methods for details. (e-f) Analysis of vitamin B12 levels in the WT and OSKM mice receiving doxycycline and the vitamin B12 bolus, as schematized in Fig. 2f. (e) Cyanocobalamin (CN-cbl) levels in the stool measured by mass spectrometry. Mice were individualized immediately after the bolus and all the fecal matter was collected from the cage 24 hours later. Untreated mice received neither the B12 bolus nor doxycycline. n = 5 mice (untreated), n = 3 (WT doxy), n = 5 (OSKM doxy), all males. (f) Vitamin B12 levels in the serum (holotranscobalamin, holoTC) measured by ADVIA Immunoassay on day 7, 24 hours after the B12 bolus, n = 5 mice (all males) per group. Color of labels indicate tissues that are prone (pancreas, colon, stomach; green) and refractory (kidney; orange) to reprogramming. Bar graphs represent average ± SD; p-values by two-tailed Student’s t-test. Source data

We next asked whether the metabolic alterations related to 1C metabolism in the serum were caused by changes specifically within those tissues undergoing reprogramming. We first examined expression of MS, which was upregulated at both the protein and RNA levels in the pancreas, colon and stomach of mice undergoing reprogramming, but not in the kidney (Fig. 2d and Extended Data Fig. 5a). Expression of *Cd320*, the main receptor for cellular uptake of B_12_ (ref. ^26^), was significantly upregulated in the reprogramming pancreas (Extended Data Fig. 5b). We also examined a gene signature of Met deprivation^39^ by gene-set enrichment analysis (GSEA). As a proof of concept, we tested this signature in previously published bulk RNA-sequencing (RNA-seq) data from in vitro OSKM reprogramming^40^, and found it was significantly enriched in iPS cells as compared to the mouse embryonic fibroblasts (MEFs) from which the iPS cells were derived (Extended Data Fig. 5c). This is consistent with the fact that ESCs require high Met levels for self-renewal and survival^34^. We then tested this Met deprivation signature in previously published bulk RNA-seq data from in vivo reprogramming^16^. At day 7 of doxycycline treatment, Met deprivation was significantly enriched in the pancreas of mice with high levels of reprogramming as compared to mice genetically resistant to reprogramming; in contrast, there was no enrichment of this pathway in the kidney (Fig. 2e). To further validate these results, we analysed a subset of genes from the Met deprivation signature, which were among the most highly enriched by GSEA, in reprogramming tissues by quantitative PCR with reverse transcription (RT–qPCR). A total of 11 genes were assessed and, interestingly, they were broadly upregulated in the pancreas, colon and stomach—but not in the kidney—of mice expressing OSKM (Extended Data Fig. 5d). A subset of these genes was basally high in the colon due to their importance in the stem cell compartment. Importantly, B_12_ supplementation generally relieved the upregulation of these genes, in support of the idea that limiting B_12_ levels were driving the Met restriction in vivo. In the kidney, these genes were induced with B_12_ supplementation, likely a feedback response caused by the large influx of B_12_ storage into the kidney after supplementation (Extended Data Fig. 3b).

Finally, to ensure that the depletion of serum B_12_ levels and its associated low levels of Met were not simply caused by defective oral uptake due to the reprogramming of several digestive organs, we administered a large bolus of vitamin B_12_ (5 µg per mouse, 100 times the recommended dietary allowance^41^) on day 6 of doxycycline treatment, 1 day before euthanasia (Fig. 2f). Mice expressing OSKM had significantly higher levels of serum B_12_ than WT mice following the bolus (Extended Data Fig. 5e,f), which is known to occur in B_12_-deficient rodents^27,31^, and further indicated that reprogramming does not compromise oral bioavailability of B_12_. Strikingly, the bolus rescued the depletion of Met levels in the serum (Fig. 2f).

Together, these data suggest that tissues undergoing reprogramming are the ones driving the depletion of serum factors that feed 1C metabolism, including Met, serine, glycine, threonine, betaine and vitamin B_12_. B_12_ becomes a limiting factor, as shown by the effects of B_12_ supplementation in rescuing Met deprivation and promoting reprogramming.

## Vitamin B_12_ plays a cell-autonomous role in reprogramming

**Fig. 3:**
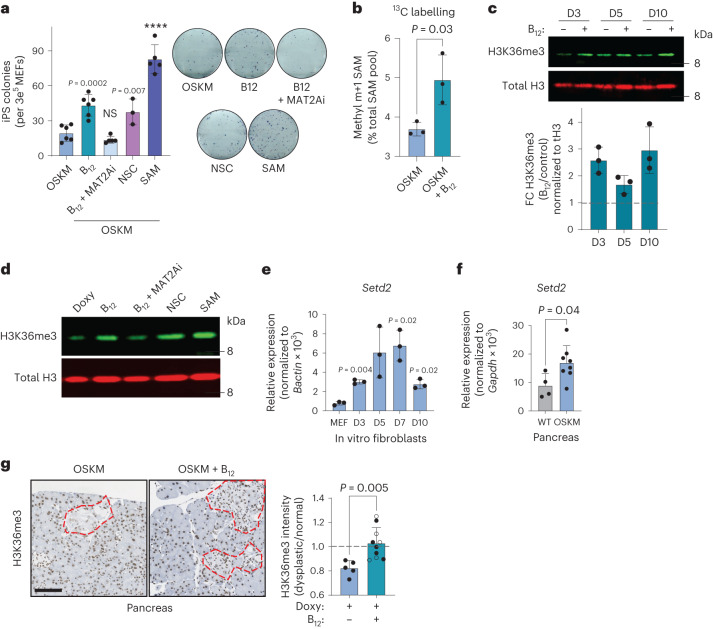
Vitamin B_12_ supplementation enhances H3K36me3 and has a cell-autonomous role in reprogramming. **a**, In vitro reprogramming of MEFs with doxycycline-inducible OSKM for 10 d in the presence of doxycycline (OSKM) and/or vitamin B_12_, MAT2Ai, KDM4A/KDM4B inhibition (NSC), or SAM as indicated, cultured in KSR. iPS cell colonies were quantified by alkaline phosphatase staining (left) and representative images are shown (right). Each data point represents MEFs generated from an independent embryo (*n* = 6 OSKM, B_12_; *n* = 5 B_12_ + MAT2Ai, SAM; *n* = 3 NSC). **b**, Fraction of total intracellular SAM ^13^C-labelled at the methyl m + 1 position (Extended Data Fig. 6d), using ^13^C-serine as a precursor. Labelling was initiated at *t* = 72 h for 6 h. Data from *n* = 3 independent MEFs are shown. **c**, H3K36me3 dynamics during in vitro reprogramming. MEFs were treated with doxycycline with or without vitamin B_12_ as indicated. A representative immunoblot and quantification from *n* = 3 independent MEFs are shown. **d**, H3K36me3 level correlates with reprogramming efficiency in vitro. MEFs were treated as indicated and H3K36me3 levels were probed in histone extracts at day 3 after doxycycline treatment. A representative blot from *n* = 2 independent MEFs with similar results is shown. **e**,**f**, Expression of the H3K36 trimethyl-transferase *Setd2* during in vitro (**e**) and in vivo pancreatic (**f**) reprogramming. In **e**, *P* values represent significant change from the parental MEFs (*n* = 3 MEFs). In **f**, samples were collected from WT (*n* = 4; 3 M 1 F) or OSKM (*n* = 8; 4 M 4 F) mice after 7 d of doxycycline treatment. **g**, H3K36me3 during in vivo reprogramming. Pancreatic tissue from OSKM mice treated with doxy (*n* = 5; 2 M 3 F) or doxy + B_12_ (*n* = 10; 4 M 6 F; Fig. 1) was stained for H3K36me3. Representative images are shown with dysplastic foci demarcated by red dashed lines. The mean nuclear optical density of the H3K36me3 stain is expressed as the ratio between the dysplastic region and the adjacent normal tissue for each mouse. Scale bar, 100 µm. Mice that received folate in addition to B_12_ are represented by open points. Graphs represent the average ± s.d.; ****P < 0.0001 by two-tailed Student’s *t*-test. Source data

**Extended Data Fig. 6:**
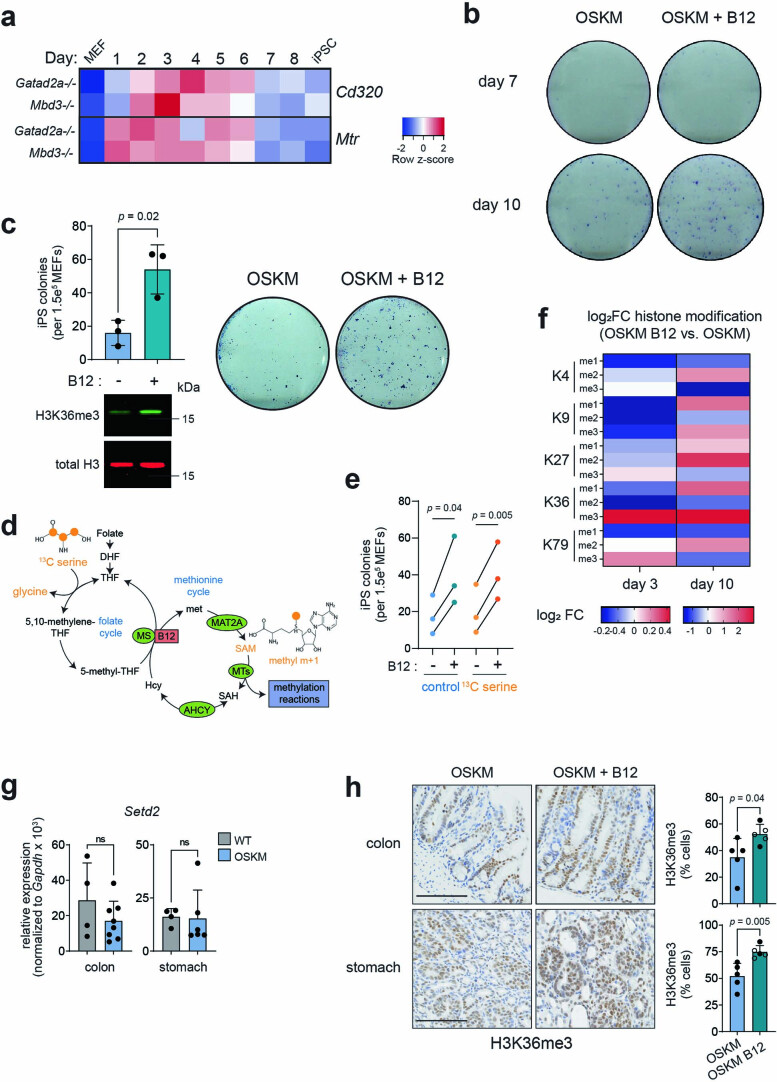
The impact of vitamin B12 administration on in vitro reprogramming. (a) Changes in gene expression of the vitamin B12 uptake receptor Cd320 and Mtr (encoding MS) during a previously published time course of iPSC generation by OSKM expression. iPSCs were derived from MEFs of two different genetic backgrounds (Gatad2a-/- and Mbd3-/) that both yield high-efficiency reprogramming. Each row is an independent embryo of the indicated genotype. Data source: 40 (b) Vitamin B12 supplementation does not accelerate iPSC formation. OSKM MEFs were treated with doxycycline with or without vitamin B12 supplementation as indicated, and iPSC colony formation was assessed by alkaline phosphatase staining after 7 and 10 days of treatment. Representative images are shown of MEFs derived from a single embryo; n = 3 independent embryos were tested. (c) Vitamin B12 enhances doxycycline-free retroviral reprogramming. Wild type MEFs were transduced with retroviral vectors encoding Oct4, Sox2, Klf4, and cMyc. Samples were collected on day 5 post-transduction for histone extraction and immunoblot (lower). iPSC colony formation was evaluated by alkaline phosphatase (AP) staining on day 14 (upper). Immunoblot and AP images are representative from n = 3 independent MEFs. (d-e) Stable isotope labeling (SIL) with fully labeled 13C-serine during in vitro reprogramming. (d) Schematic of 1 C metabolism indicating how fully labeled 13C-serine contributes to methyl m + 1 labeling of SAM. Orange points indicate 13 C atoms. Figure adapted from ref. ^33^, Springer Nature Limited. (e) SIL does not affect reprogramming efficiency. Doxycycline-inducible OSKM MEFs were cultured in normal iPS media with doxycycline without or without vitamin B12 supplementation as indicated. 13C-serine labeled media was administered as descried in Methods and reprogramming was quantified by iPS colonies evaluated by AP staining on day 10. Quantification of colonies is shown for the indicated treatments. Each point represents an individual MEF clone. P-values represent comparison of the OSKM vs OSKM + B12 condition for each media type. (f) Changes in histone methylation during in vitro reprogramming with vitamin B12 supplementation. Histone extracts were prepared from OSKM MEFs treated with doxycycline for 3 and 10 days, with or without vitamin B12 supplementation. Levels of the indicated histone methylation modifications were measured by colorimetric multiplex assay and the relative abundance (fold change, FC) in cells reprogrammed in the presence as compared to absence of vitamin B12 is shown. Note that MEFs derived from independent embryos were used for day 3 and day 10 analyses. (g) Expression of Setd2, which encodes the H3K36 trimethyl-transferase in the colon (left) and stomach (right) from WT and OSKM mice after 7 days of doxycycline treatment. Mice are from Fig. 1b n = 4 mice (WT), n = 8 (OSKM colon), n = 6 (OSKM stomach). (h) H3K36me3 during in vivo reprogramming in the colon and stomach of OSKM mice with and without B12 supplementation. Representative images are shown (left) and quantified (right) in a subset (n = 5 for each group) of mice from Fig. 1e. Scale bar is 100 µm. Mice that received folate in addition to B12 are represented by open points. Graphs represent average ± SD. P-values by two-tailed Student’s t-test. used for day 3 and day 10 analyses. (g) Expression of Setd2, which encodes the H3K36 trimethyl-transferase in the colon (left) and stomach (right) from WT and OSKM mice after 7 days of doxycycline treatment. Mice are from Fig. 1b n = 4 mice (WT), n = 8 (OSKM colon), n = 6 (OSKM stomach). (h) H3K36me3 during in vivo reprogramming in the colon and stomach of OSKM mice with and without B12 supplementation. Representative images are shown (left) and quantified (right) in a subset (n = 5 for each group) of mice from Fig. 1e. Scale bar is 100 µm. Mice that received folate in addition to B12 are represented by open points. Graphs represent average ± SD. P-values by two-tailed Student’s t-test. Source data

In vivo, vitamin B_12_ deficiency yields a complex phenotype because it impacts multiple cellular processes and organ functions^26^. Therefore, we asked whether the effect of B_12_ on reprogramming could be recapitulated in vitro. We first observed, in a previously published bulk RNA-seq dataset of MEFs undergoing in vitro reprogramming^40^, that *Mtr* and *Cd320* were upregulated soon after OSKM induction, remaining high during the early and middle phases of reprogramming, ultimately stabilizing to levels above those measured in MEFs (Extended Data Fig. 6a). This suggests that during in vitro reprogramming there is also a high demand of B_12_ and Met, which we explored further using pharmacological manipulation of the Met cycle. The addition of B_12_ significantly increased the efficiency of iPS cell colony formation (Fig. 3a), recapitulating our observations in vivo and demonstrating a cell-intrinsic role for vitamin B_12_ in reprogramming. Of note, B_12_ supplementation increased the number of successfully formed iPS cell colonies without an obvious effect on the rate of colony formation (Extended Data Fig. 6b), and the improved efficiency was also observed in a doxycycline-free, retroviral-based reprogramming system in WT MEFs (Extended Data Fig. 6c). The B_12_-mediated increase in reprogramming efficiency was prevented by concomitant treatment with a methionine adenosyltransferase 2A inhibitor (MAT2Ai; Fig. 3a); MAT2A is the enzyme immediately downstream of MS, which converts Met into SAM (Fig. 2a). Moreover, directly supplementing only SAM at a high concentration^34^ during reprogramming significantly improved the efficiency of the process, even beyond that of B_12_ itself (Fig. 3a).

To directly assess the contribution of B_12_ supplementation to SAM generation, we performed stable isotope labelling (SIL) with ^13^C-labelled serine. Serine significantly decreases in the serum during in vivo reprogramming (Extended Data Fig. 4b) and can contribute as a methyl donor to 1C metabolism (Extended Data Fig. 6d). We began SIL 72 h after OSKM induction, well before iPS cell colonies are formed. Culturing cells with ^13^C-serine did not affect the reprogramming efficiency, nor the capacity of B_12_ to enhance reprogramming (Extended Data Fig. 6e). Importantly, B_12_ significantly stimulated the incorporation of the ^13^C-methyl donor group (m + 1) from serine into SAM (Fig. 3b). Collectively, these data demonstrate that B_12_ operates in a cell-intrinsic manner during in vitro reprogramming and that it is a limiting factor for SAM generation and successful reprogramming in vitro.

## H3K36me3 is enhanced by vitamin B_12_ during reprogramming

Among the SAM-dependent cellular processes, histone methylation is one of the highest consumers of SAM^42,43^. Histone modifications play a major role in determining cellular identity changes during reprogramming^44,45^, and SAM levels are critical for both mouse^12^ and human^34^ embryonic stem cell maintenance through appropriate histone modifications. Moreover, some histone modifying enzymes have an affinity for SAM that is in the physiological range of this metabolite and, therefore, their enzymatic output is sensitive to changes in SAM levels^42^. This led us to hypothesize that vitamin B_12_, through SAM, could promote changes in histone methylation during reprogramming. To identify B_12_-mediated histone methylation changes in an unbiased manner, we performed a histone H3 modification array at both early (day 3) and late (day 10) stages of the reprogramming process in vitro. While there were many epigenetic changes associated with vitamin B_12_ supplementation, trimethylated histone H3 Lys 36 (H3K36me3) was the histone modification that increased the most, and the only one that increased at both day 3 and day 10 of OSKM induction in MEFs (Extended Data Fig. 6f). We used immunoblotting to confirm the results of the histone array and found that H3K36me3 was enhanced by the addition of vitamin B_12_ during reprogramming at all time points tested (Fig. 3c). B_12_ also enhanced H3K36me3 in MEFs undergoing reprogramming via retroviral OSKM, in the absence of doxycycline (Extended Data Fig. 6c). H3K36me3 levels correlated with the ability of 1C modulators to promote reprogramming efficiency: MAT2Ai reduced the amount of H3K36me3, while SAM enhanced it (Fig. 3d). We also tested NSC636819 (NSC), an inhibitor of the H3K36me3 (and H3K9me3) demethylases KDM4A/KDM4B^46^. In the absence of B_12_ supplementation, NSC enhanced both H3K36me3 levels (Fig. 3d) and reprogramming efficiency to the level of cells treated with B_12_ alone (Fig. 3a).

We then tested if the link between B_12_, H3K36me3 and reprogramming observed during in vitro reprogramming of MEFs could also be substantiated during in vivo reprogramming. First, we found that the mRNA levels of *Setd2*, the gene encoding the only known mammalian methyltransferase that catalyses H3K36 trimethylation^47^, were significantly increased during both in vitro reprogramming of MEFs (Fig. 3e) and in vivo reprogramming in the pancreas (Fig. 3f). Immunohistochemistry revealed that the regions of the exocrine pancreas undergoing reprogramming (identified by their abnormal architecture) had reduced levels of H3K36me3 as compared to the surrounding tissue (with normal histology; Fig. 3g). Importantly, in vivo supplementation with B_12_ restored H3K36me3 levels within dysplastic regions to parity with those of the neighbouring normal tissue (Fig. 3g). Although we did not detect changes in *Setd2* expression in the colon and stomach with reprogramming (Extended Data Fig. 6g), B_12_ also significantly increased H3K36me3 levels in these tissues (Extended Data Fig. 6h), indicating that the SETD2 enzymatic activity is sensitive to B_12_ levels. Collectively, these data strongly suggest that B12 levels are mechanistically linked to H3K36me3 levels, and that both are positively correlated with the efficiency of reprogramming in vitro and in vivo.

## Vitamin B_12_ improves transcriptional fidelity during reprogramming

**Fig. 4:**
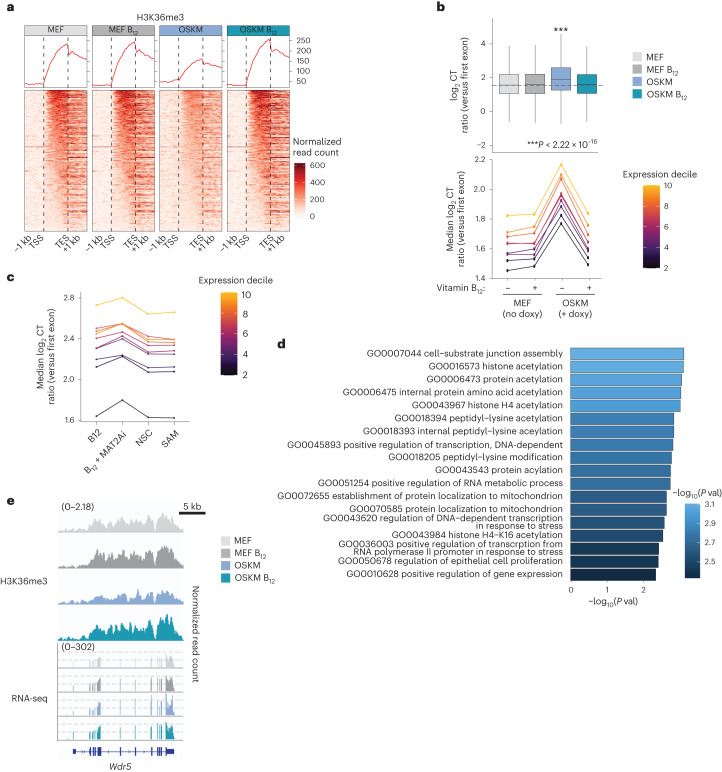
Vitamin B_12_ supplementation reduces cryptic transcription during in vitro reprogramming. **a**, H3K36me3 ChIP–seq was performed in MEF 1 (Extended Data Fig. 7) at 72 h after the addition of doxycycline and/or vitamin B_12_, as indicated. The normalized ChIP reads in gene bodies relative to all aligned reads are shown for the most highly expressed genes (top tercile). The trace of the ChIP signal is shown, where the *y* axis represents average coverage across genes. TSS, transcription start site; TES, transcription end site. See also Extended Data Fig. 7c. **b**, The level of CT in MEF 1 for all genes for which a score could be calculated is plotted globally, and as a function of gene expression level at *t* = 72 h, with or without continuous supplementation of vitamin B_12_ as indicated. The lower and upper hinges correspond to the first and third quartiles (the 25th and 75th percentiles). The upper and lower whiskers extend from the hinge to the largest and smallest values, respectively, no further than 1.5 times the interquartile range from the hinge. Asterisks represent two-tailed unpaired Wilcoxon test; no multiple-comparisons adjustment was performed. **c**, OSKM MEFs were cultured with doxycycline and additional compounds for 72 h as indicated and described in Fig. 3a. The median CT ratio for all genes for which a score could be calculated is plotted as a function of gene expression level. Values represent the median from *n* = 2 independent MEFs; for data from individual MEFs, see Extended Data Fig. 7e. **d**, Functional enrichment analysis was performed on the top 25% of genes whose CT increased between MEF and OSKM conditions, and decreased between OSKM and OSKM + B_12_ conditions in MEF 1 from **a** and **b**. The top enriched GO biological processes (*P* < 0.005) are shown. *P* values were determined by hypergeometric test; no multiple-comparisons adjustment was performed. **e**, H3K36me3 ChIP–seq and RNA-seq tracks from the *Wdr5* gene from MEF 1 in the indicated conditions. For all plots, expression quantiles were determined by reads per kilobase per million mapped reads (RPKM) across all samples. Source data

**Extended Data Fig. 7:**
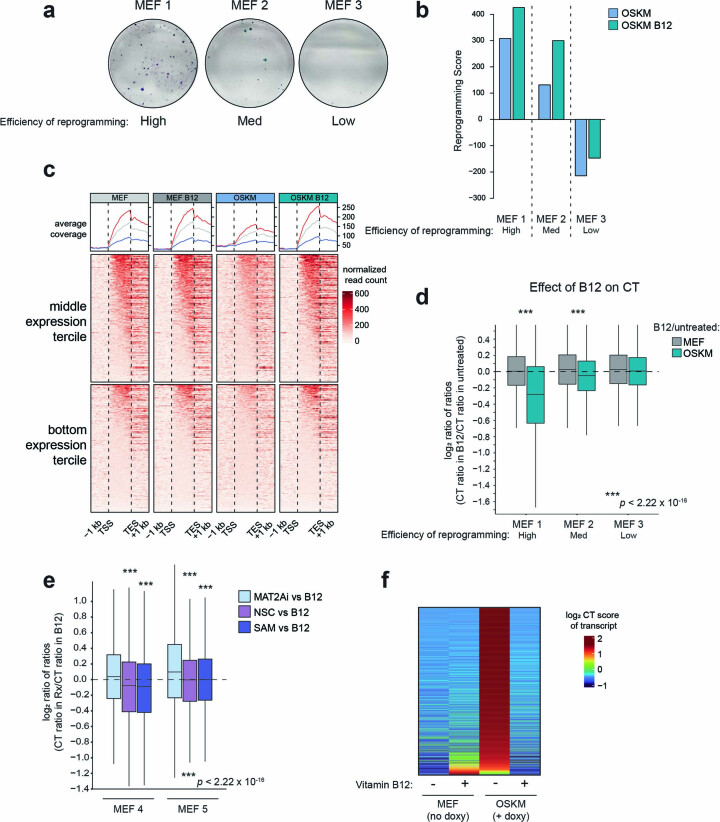
Reprogramming and transcriptional dynamics across MEF clones. (a) The reprogramming efficiency of three independent MEFs was measured by the formation of iPS colonies as stained with alkaline phosphatase 10 days after continuous addition of doxycycline to the media. (b) The Reprogramming Score was calculated using transcriptional data from MEFs expressing doxycycline-inducible OSKM for 72 h, whose cell surface markers indicated they were poised for high-efficiency reprogramming^48^. See Methods for details. (c) H3K36me3 chromatin immunoprecipitation (ChIP) sequencing was performed in Clone 1, 72 h after the addition of doxycycline and/or vitamin B12, as indicated. The normalized ChIP reads in gene bodies relative to all aligned reads are shown for the middle and bottom tercile in terms of gene expression (as determined by RNA-seq RPKM levels across all condition) are shown (lower). The trace of the ChIP signal is shown, where the y-axis represents average coverage across genes (upper; blue = bottom tercile; grey = middle tercile; red = top tercile, shown for reference; see Fig. 4a for top tercile). TSS – transcription start site; TES – transcription end site. (d-e) The ratio of cryptic transcription (CT). (d) CT in B12-treated cells as compared to non-B12 is shown for the median CT ratio for all genes for which a score could be calculated is plotted. Individual MEFs (n = 3 total) are shown, separated into MEF (no doxycycline) and OSKM (72 h of doxycycline treatment to induce OSKM expression) conditions. Asterisks represent the comparison between the MEF and OSKM bars. (e) OSKM cells were treated with doxycycline and the indicated compounds for 72 h, as described in Fig. 4c. The ratio of CT is plotted as a ratio of the CT in the B12 condition for each treatment, in n = 2 independent MEFs. Asterisks represent the comparison between the indicated treatment and the MAT2Ai treatment. The lower and upper hinges correspond to the first and third quartiles (the 25th and 75th percentiles). The upper (lower) whisker extends from the hinge to the largest (smallest) value no further than 1.5 * IQR from the hinge. P-values were determined by two-tailed unpaired Wilcoxon test no multiple comparisons adjustment (f) Genes whose CT increases upon reprogramming (top 25% comparing OSKM vs. MEF) and decrease with vitamin B12 supplementation (top 25% comparing OSKM B12 vs. OSKM) in Clone 1, used for the hypergeometric analysis in Fig. 4d. The CT ratio of individual genes is shown for each condition. Source data

To address the role of H3K36me3 during reprogramming, we performed (i) chromatin immunoprecipitation followed by sequencing (ChIP–seq) for H3K36me3 and (ii) bulk RNA-seq in MEFs undergoing reprogramming, with and without supplementation of vitamin B_12_. We chose to analyse reprogramming at 72 h because *Setd2* mRNA is already significantly upregulated (Fig. 3e) and B_12_ supplementation has increased global H3K36me3 levels (Fig. 3c) at this time point, but iPS cell colonies have not yet formed. After collecting the 72-h samples, we allowed the MEFs from three independent embryos to continue reprogramming up to 10 days, when iPS cells can be seen by light microscopy and stain positive for alkaline phosphatase. We observed that the three MEFs exhibited different reprogramming efficiencies: MEF 1 > MEF 2 > MEF 3 (Extended Data Fig. 7a). This differential efficiency of reprogramming was also reflected in the transcriptomes of these cells, as evaluated by the enrichment of a reprogramming score composed of genes previously reported in MEFs that are ‘poised to reprogram’ at 72 h of OSKM expression^48^ (Extended Data Fig. 7b). We focused our subsequent analyses on MEF 1 because of its higher reprogramming efficiency. Consistent with published ChIP data from yeast^49^, mouse^50,51^ and human cells^52^, we found the H3K36me3 ChIP signal largely distributed within gene bodies, peaking towards the 3′ ends (Fig. 4a and Extended Data Fig. 7c). Interestingly, 72 h of OSKM induction caused a decrease in the amount of H3K36me3 across gene bodies relative to all reads, which was prevented by vitamin B_12_ supplementation (Fig. 4a and Extended Data Fig. 7c).

H3K36me3 plays important roles in maintaining transcriptional fidelity^49,52–56^. One such mechanism, active in mammalian stem cells, is to prevent illegitimate transcription initiation outside of promoter regions, at intragenic ‘cryptic’ start sites, particularly within genes that are highly expressed^50,52^. We thus asked whether the amount of cryptic transcription (CT) was changed during reprogramming. CT can be estimated by calculating the ratio of expression from 3′ exons (defined as those from the fourth to the penultimate exon) over the expression from the first exon^50^ (Supplementary Table 4 and Methods). Consistent with the reduced levels of H3K36me3, global CT significantly increased during OSKM-induced reprogramming as compared to the non-induced MEFs, across genes of all expression levels (Fig. 4b). Remarkably, supplementation with vitamin B_12_ during these 72 h of OSKM expression reduced the amount of CT to basal (that is, non-reprogramming) levels (Fig. 4b). This effect was dose dependent with respect to the level of reprogramming in each MEF (Extended Data Fig. 7d). Vitamin B_12_ treatment in the non-induced MEFs did not affect CT (Fig. 4b and Extended Data Fig. 7d). These data suggest that the high demand for methylation that occurs during reprogramming causes a deficit in H3K36me3 that results in increased CT, which can be rescued by B_12_ supplementation. To further substantiate this concept, we measured reprogramming-associated CT in the presence of pharmacological modulators that aggravate the methylation deficit (MAT2Ai), that alleviate the methylation deficit (SAM), and that stabilize H3K36me3 (NSC). Interestingly, MAT2Ai increased, while SAM and NSC decreased reprogramming-associated CT (Fig. 4c and Extended Data Fig. 7e).

We next asked whether the genes that suffered reprogramming-associated CT had a specific function. We used a hypergeometric test against GO gene-set collections to analyse the function of the top 25% of genes whose CT was induced by OSKM expression, and whose CT could be reduced with vitamin B_12_ (Extended Data Fig. 7f). We found that, together with cell-substrate junction assembly, the most highly enriched GO categories were related to histone modification and transcriptional control (Fig. 4d), suggesting that B_12_ helps to streamline transcription during reprogramming. In support of this, the transcriptional reprogramming score of cells poised to reprogram^48^ was improved by B_12_ supplementation (Extended Data Fig. 7b). *Wdr5*, a gene whose expression is important for the establishment of pluripotency^57^, was among those genes whose CT was most largely changed, and whose ChIP peaks exemplify these H3K36me3 and CT dynamics during reprogramming (Fig. 4e). Overall, we found that during the early stages of reprogramming, cells undergo a depletion of H3K36me3 along gene bodies that compromises transcriptional fidelity, as reflected by a global increase in CT from expressed genes. Supplementation of vitamin B_12_ restores H3K36me3 levels and suppresses CT, improving the reprogramming trajectory of the bulk population.

## Vitamin B_12_ promotes tissue repair after DSS-induced colitis

**Fig. 5:**
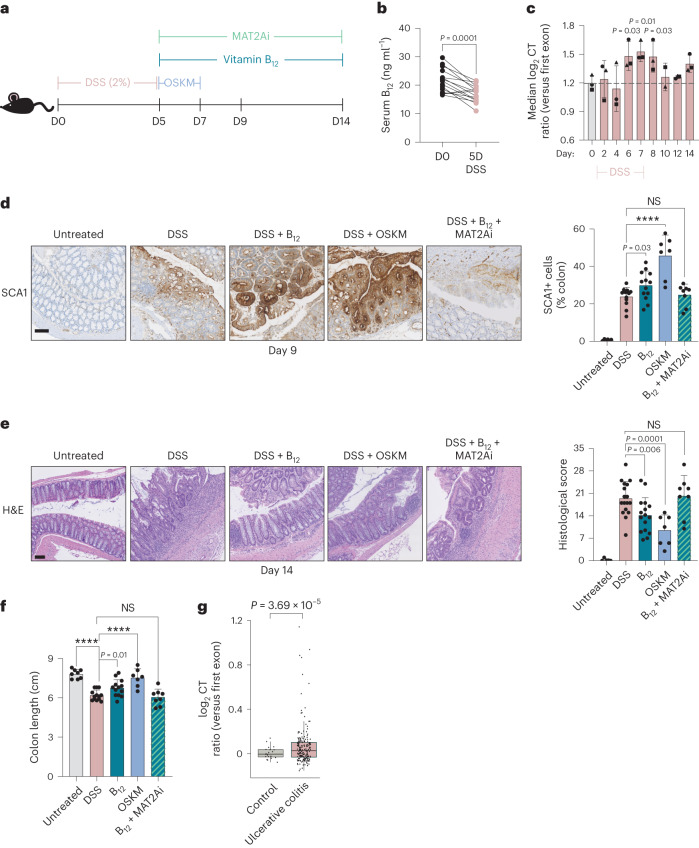
Recovery from DSS colitis is improved by OSKM or vitamin B_12_. **a**, Colitis was induced by DSS, followed by a 48-h pulse of OSKM or continuous administration of vitamin B_12_ during recovery, as indicated. MAT2Ai is FIDAS-5, a MAT2A inhibitor, given daily over the indicated time. **b**, Immunoassay of serum holoTC (biologically available vitamin B_12_) in paired mice before (D0) or following (D5) DSS administration (*n* = 15; 15 M); serum was collected repeatedly from the same mice. *P* value was determined by paired two-tailed Student’s *t*-test. **c**, CT in mouse colon tissue during DSS injury and recovery was determined from previously published RNA-seq. Data source: GSE131032 (ref. ^62^). The median CT ratio for all genes for which a score could be calculated is shown. Each time point has 2–3 biological replicates, and symbol shapes indicate the same cage, as reported by the authors. *P* values were compared to the median CT at D0 as computed by two-tailed Student’s *t*-test; dashed line represents median CT value at D0. See also Extended Data Fig. 8a. **d**, SCA1 immunohistochemistry in colonic sections on day 9 after DSS with the indicated treatments. Representative images are shown and percentage of SCA1^+^ tissue area is quantified. Untreated control (*n* = 6; 3 M 3 F); DSS (*n* = 12; 8 M 4 F); DSS + vitamin B_12_ (*n* = 13; 9 M 4 F); DSS + 48 h OSKM (*n* = 7; 7 M); DSS + B_12_ + MAT2Ai (*n* = 8; 4 M 4 F). Graphs represent four pooled experiments, each of which had at least *n* = 3 each DSS and DSS + B_12_ controls. *P* values represent the difference as compared to DSS control animals by one-way ANOVA. **e**, Recovery of colonic homeostasis as scored by H&E on day 14 after DSS with the indicated treatments (representative images and quantification). Untreated control (*n* = 5; 5 M); DSS (*n* = 17; 11 M 6 F); DSS + vitamin B_12_ (*n* = 16; 10 M 6 F); DSS + 48 h OSKM (*n* = 7; 4 M 3 F); DSS + B_12_ + MAT2Ai (*n* = 8; 4 M 4 F). Graphs represent five pooled experiments, each of which had at least *n* = 3 each DSS and DSS + B_12_ controls. *P* values represent difference as compared to DSS control animals by one-way ANOVA. **f**, Colon length (caecum to rectum) on day 14 after DSS from mice with the indicated treatments. Untreated control (*n* = 8; 3 M 5 F); DSS (*n* = 13; 9 M 4 F); DSS + vitamin B_12_ (*n* = 13; 10 M 3 F); DSS + 48 h OSKM (*n* = 7; 4 M 3 F); DSS + B_12_ + MAT2Ai (*n* = 8; 4 M 4 F). *P* values as compared to DSS control animals by one-way ANOVA. **g**, CT in previously published RNA-seq of paediatric human rectal mucosal biopsy samples classified as ulcerative colitis (*n* = 206; 112 M 94 F) or control (*n* = 20; 9 M 11 F). Data source: GSE109142 (ref. ^63^). Median CT value for all genes for which a score could be calculated is shown, where each dot represents one patient. The lower and upper hinges correspond to the first and third quartiles (the 25th and 75th percentiles), respectively. The upper (lower) whisker extends from the hinge to the largest (smallest) value no further than 1.5 times the interquartile range from the hinge. Data beyond the end of the whiskers are called ‘outlying’ points and are plotted individually. *P* value calculated by linear model with sex and quantiles as covariables; no multiple-comparisons adjustment was performed. Scale bars, 100 µm. Bar graphs represent the average ± s.d. ****P < 0.0001. Source data

**Extended Data Fig. 8:**
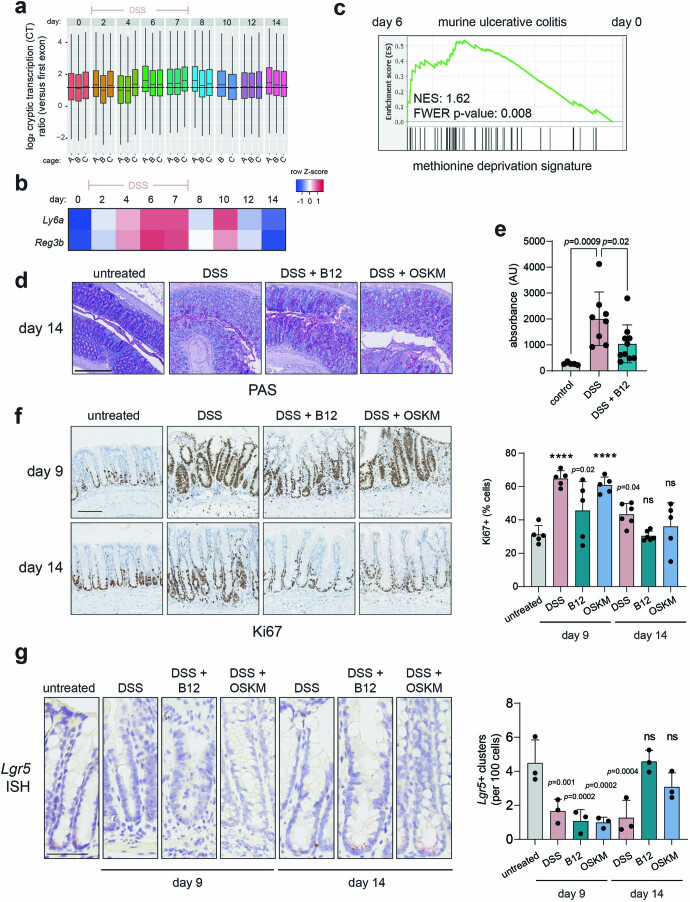
Recovery from DSS colitis is improved by OSKM or vitamin B12. (a-c) Previously published time course of bulk colonic RNAseq during DSS injury and recovery Data source: GSE131032^63^. Mice were treated with 2.5% DSS for 7 days as indicated. (a) CT analysis for all genes for which a score could be calculated are shown. Each boxplot represents one biologic replicate (that is, mouse), with the cages indicated as described by the authors of the original study. Dashed line represents median CT value at D0. (b) RNA-sequencing expression of the indicated genes. Values represent the scaled log2FC as compared to D0 across all samples as computed by the authors of the original study. (c) Gene set enrichment analysis of a published signature of methionine deprivation (msigdb: M13537 ref. ^39^) in samples from day 6 vs day 0 of DSS treatment as computed by the authors of the original study. (d-g) Recovery from DSS injury of mice in Fig. 5, with treatment as schematized in Fig. 5a; a representative subset of mice was used for analyses. (d) Periodic acid–Schiff (PAS) staining, which marks mucous produced by differentiated goblet cells, in colonic sections of mice on day 14. n = 5 mice from each group were analyzed with similar results; representative images are shown. (e) Intestinal barrier permeability as measured by FITC-dextran leak assay. Fluorescence intensity was measured in the serum of mice with the indicated treatments on day 9. n = 5 mice (control), n = 8 (DSS), n = 10 (DSS + B12). (f) Ki67 was quantified within the colonic epithelium (right) and representative immunohistochemistry images are shown (left). P-values indicate comparison to the untreated control. Scale bar is 100 µm. n = 6 mice (DSS D14), n = 5 all other groups. (g) Analysis of Lgr5 by single molecule in situ hybridization (ISH). The number of Lgr5+ clusters per 100 cells in the colonic crypts was quantified from at least 20 crypts per mouse (right) from n = 3 mice and representative images are shown (left). Asterisks indicate comparison to the untreated control. Scale bar is 50 µm. Bar graphs represent average ± SD; ****p < 0.0001 by one-way ANOVA. Source data

Many processes of tissue injury and repair in adult organs proceed through transient dedifferentiation to a more developmentally primitive state^22,58^. Moreover, transient cell plasticity achieved through OSKM reprogramming has been shown to promote regeneration after acute injury in several organs including the pancreas, muscle^4^, eye^6^ and heart^7^. We reasoned that vitamin B_12_ administration during an injury repair period may also promote cell plasticity and improve recovery. To address this idea, we used a dextran sodium sulfate (DSS) model of acute ulcerative colitis (Fig. 5a). In this model, successful repair proceeds through natural reprogramming of epithelial cells to a more embryonic-like state, marked by the murine stem cell marker *Ly6a* (encoding SCA1)^22,59,60^. Of note, this marker of tissue repair is also characteristic of the intermediate, plastic state of OSKM reprogramming in vivo in the colon, as well as in the stomach and the pancreas^19^. Interestingly, we found that DSS injury, much like OSKM reprogramming, was associated with a significant depletion of serum vitamin B_12_ levels (Fig. 5b). It is important to note that this depletion is unlikely to be attributed to colitis-associated nutrient absorption defects, as B_12_ is typically absorbed in the terminal ileum^26^, while DSS primarily affects the distal colon^61^. We asked whether this pathophysiological example of cellular plasticity and B_12_ deficiency was also associated with increased CT using a previously published time-course analysis of bulk RNA-seq of the murine colon during DSS injury and repair^62^. We found that colonic CT significantly increased on days 6, 7 and 8 following DSS treatment initiation (Fig. 5c and Extended Data Fig. 8a), concomitant with the transcriptional peak of tissue repair markers like *Ly6a* and *Reg3b*^22^ (Extended Data Fig. 8b). We also observed a significant enrichment of the Met deprivation signature on day 6, as compared to uninjured mice (Extended Data Fig. 8c).

We next asked whether we could enhance or accelerate tissue repair. Following 5 days of DSS administration, we treated recovering mice either with a brief (48 h) pulse of OSKM expression, or with continuous vitamin B_12_ supplementation (Fig. 5a). On day 9, 4 days after withdrawal of DSS, we confirmed the previously reported expression of SCA1 in the repairing epithelium, which was further increased with either the OSKM pulse or B_12_ supplementation (Fig. 5d). Thus, both approaches could promote tissue reprogramming in the context of acute colitis. Remarkably, both OSKM and B_12_ resulted in significantly improved tissue recovery on day 14, as evaluated by blinded histological analysis (Fig. 5e), colon length (Fig. 5f) and mucosal integrity as visualized by PAS staining (Extended Data Fig. 8d). We used fluorescein isothiocyanate-conjugated (FITC) dextran leakage to evaluate functional recovery of intestinal permeability after B_12_ administration, and found it was also significantly improved (Extended Data Fig. 8e). Vitamin B_12_ and OSKM treatment also accelerated the recovery towards normal homeostatic colonic crypt architecture, as measured by the number of Ki67^+^ cells (Extended Data Fig. 8f) and the recovery of *Lgr5* expressing cells (Extended Data Fig. 8g). The increase in SCA1 and the enhanced recovery promoted by B_12_ were blunted by the co-administration of a MAT2A inhibitor (FIDAS-5), suggesting that SAM generation is critical for tissue repair (Fig. 5d,e). To extend our findings to a comparable human pathology, we found CT was significantly increased in a previously published bulk RNA-seq dataset^63^ of paediatric ulcerative colitis patients as compared to healthy controls (Fig. 5g), suggesting a conserved mechanism in human disease. Thus, echoing our findings during OSKM-driven reprogramming, tissue repair is associated with depletion of B_12_ and high levels of CT, and vitamin B_12_ supplementation facilitates tissue repair.

## Discussion

In this study, we analysed the murine faecal microbiota during reprogramming to discover that vitamin B_12_ is a limiting factor for cellular reprogramming both in vitro and in vivo. We demonstrate that cells undergoing reprogramming experience an elevated demand for vitamin B_12_, associated with increased levels of the B_12_-dependent enzyme MS and increased expression of *Cd320*, which encodes the cellular vitamin B_12_ uptake receptor. MS functions to generate Met, which is converted into SAM, the essential 1C donor for all methylation reactions^64^. We report that SAM is central to the improved reprogramming efficiency imparted by vitamin B_12_. One-carbon metabolism and SAM, via its donation of methyl groups, are required for the epigenetic reconfiguration associated with cell reprogramming^45^. Mechanistically, we found that insufficiency of vitamin B_12_ during OSKM reprogramming resulted in changes in the bulk levels of many histone methylation marks, including a failure to properly trimethylate H3K36 at transcribed gene bodies, leading to increased levels of CT. CT has negative impacts on cellular and organismal fitness^49,55^ and compromises the self-renewal capacity of mammalian stem cells^52^. While we could not directly test the role of H3K36me3 via *Setd2* knockdown because we were technically unable to achieve sustained transcript reduction, supplementation of vitamin B_12_ increased H3K36me3 and reduced CT during reprogramming, associated with an enhanced transcriptional reprogramming score and an overall increase in reprogramming efficiency.

It has been estimated that the complete methylation of just 0.1% of nucleosomes would exhaust the entire supply of intracellular SAM^65,66^, rendering the epigenome a critical consumer of SAM levels. SETD2, the H3K36me3 methyltransferase, has a *K*_M_ for SAM in the micromolar range^67^, close to the physiological intracellular concentration of SAM. This makes SETD2 particularly sensitive to fluctuations in intracellular SAM levels, and may explain why we found this particular H3 mark to be the most increased after B_12_ supplementation.

Nevertheless, we suspect that the role of B_12_ in induced plasticity extends beyond H3K36me3 and CT regulation. Levels of histone methylation are dynamic and inter-dependent^43^. SAM has been described as one of the few ‘sentinel metabolites’ of the cell, with an estimated 1% of all eukaryotic proteins being SAM-dependent methyltransferases with diverse substrates^65,66^. It will be interesting to explore how the additional histone H3 methyl changes we observed with B_12_ supplementation, as well as yet-unidentified changes in methylation of DNA, other proteins, or lipids may contribute to the improved reprogramming efficiency achieved by vitamin B_12_.

In vivo, we report that depletion of the microbiota with broad-spectrum antibiotics profoundly limits reprogramming. This was only partially rescued by supplementation of B_12_, indicating that the microbiota plays additional roles in driving this process. We suspect that one such role is enhancing inflammation, particularly IL-6 production, which is critical for in vivo reprogramming^16^. Interestingly, we also found that the microbiota is important for oral bioavailability of doxycycline beyond the gastrointestinal tract, highlighting the complex relationship between commensal bacteria, doxycycline-inducible systems and in vivo reprogramming. We took advantage of in vitro reprogramming systems, including doxycycline-free viral reprogramming, to directly demonstrate the limiting, cell-intrinsic role of B_12_ in this process. Nevertheless, it will be interesting to further study the relationships between B_12_, metabolism, microbiota and doxycycline in vivo.

From a biomedical perspective, we have translated our results into a pathological disease model of acute ulcerative colitis, in which intestinal epithelial repair requires dedifferentiation of enterocytes to an embryonic-like state^22^. We have shown that further increasing this dedifferentiation is associated with improved colonic tissue repair, and that vitamin B_12_ alone can facilitate this process. In future studies, it would be valuable to perform a denser time-course analysis in the presence of vitamin B_12_ to refine the molecular details of the enhanced repair process. Prior studies have reported a protective role of chronic B_12_ deficiency (that is, before DSS administration); this was due to a blunted inflammatory response to the DSS insult^68,69^. Indeed, B_12_ deficiency can have immunomodulatory properties^70^, and it will be interesting to study the impact of acute, post-injury supplementation of B_12_ on the immune compartment.

We have also shown increased CT in both murine and human samples of ulcerative colitis, suggesting further similarities between OSKM and tissue repair. Consistent with this, *Setd2* deletion in intestinal epithelial cells exacerbates DSS injury, and its expression is lost in a high proportion of individuals with human inflammatory bowel disease^71^. In human inflammatory bowel disease, there is evidence of B_12_ deficiency^72^, and polymorphisms in the vitamin B_12_ transporter transcobalamin II have been associated with ulcerative colitis^73^. These findings suggest a therapeutic role for B_12_ in human disease.

We find it intriguing that there is common upregulation of SCA1 during the intermediate, plastic, phase of OSKM-driven reprogramming^19^ and during multiple processes of somatic tissue repair, including the intestine^22,59,60^, pancreas^74^, kidney^75,76^ and lung^77,78^. We speculate that similar plasticity-mediated processes are conserved across various adult tissues of limited regenerative capacity, and that vitamin B_12_ may also improve repair in these diverse settings. Overall, our results advance our current molecular understanding of in vivo and in vitro reprogramming and highlight the possibility to safely administer B_12_ for the therapeutic enhancement of cellular plasticity for regenerative medicine, organ injury and repair.

## Methods

### Animal procedures

Animal experimentation at the IRB Barcelona was performed according to protocols approved by the Science Park of Barcelona (PCB) Ethics Committee for Research and Animal Welfare. Mice were housed in a specific pathogen-free facility on a 12-hour light–dark cycle at an ambient temperature of 20–24 °C and humidity of 30–70%. Adult mice were fed ad libitum with SAFE R40 pellet diet (https://safe-lab.com/safe_en/) containing 0.02 mg per kg body weight vitamin B_12_. In general, mice of 8–16 weeks of age of both sexes were treated with 1 mg ml^−1^ doxycycline hyclate BioChemica (PanReac, A2951) in the drinking water (supplemented with 7.5% sucrose) for 7 d. Antibiotic treatment was conducted using a broad-spectrum cocktail (1 mg l^−1^ each of ampicillin (BioChemica, A0839), neomycin sulfate and metronidazole (Sigma, M1547); 0.5 mg l^−1^ vancomycin (Cayman Chemical, CAY-15327) all dissolved in water supplemented with 7.5% sucrose) for 3 weeks before doxycycline initiation and was maintained during doxycycline treatment. Vitamin B_12_ (Sigma, V2876) supplementation was provided at 1.25 mg l^−1^ and folate supplementation was provided as folic acid (Sigma, F7876) at 40 mg l^−1^ in the drinking water, both for 7 d concomitant with doxycycline treatment. For the B_12_ bolus experiment, mice were administered 5 µg vitamin B_12_ (Sigma, V2876) dissolved in water by oral gavage on day 6 after the start of doxycycline treatment, and blood samples were taken by submandibular collection just before and 24 h after the bolus. OSKM transgenic mice are the i4F-B strain (derived on a C57/BL6J background and bred in house) described in ref. ^3^ and are available upon request. WT mice were i4F-B WT littermate controls where specified, or WT C57/BL6J (Charles River France).

#### DSS-induced colitis

Mice were treated with 2.5% (wt/vol) DSS, colitis grade (36,000–50,000; MP Biomedicals, MFCD00081551) in drinking water for 5 consecutive days. On day 5, the DSS was removed and drinking water was supplemented with doxycycline hyclate BioChemica (1 mg ml^−1^; PanReac, A2951; with 7.5% sucrose) for 48 h, after which regular water was returned. Mice in the B_12_ experimental group also received supplementation of vitamin B_12_ (1.25 mg l^−1^; Sigma, V2876) from the point of DSS removal (that is, day 5) until experimental endpoint. The MAT2Ai group received FIDAS-5 (MedChemExpres, HY-136144) and were dosed with 20 mg per kg body weight per day dissolved in PEG400 by oral gavage as previously described^79^.

#### FITC–dextran intestinal permeability assay

On day 9 (relative to the start of DSS administration), food was withdrawn from mice for 4 h, after which mice were gavaged with FITC–dextran (MW 4,000; Sigma-Aldrich, FD4) at a dose of 44 mg per 100 g of body weight dissolved in PBS. Food restriction was maintained for 3 additional hours, at which point blood was sampled by submandibular vein bleeding. Whole blood was diluted at a ratio of 1:4 in PBS, and 100 µl of blood/PBS mixture from each mouse was loaded into a 96-well plate. Fluorescence intensity was measured on a BioTek Synergy H1 Microplate Reader (excitation 490 nm; emission 520 nm).

### Microbial analysis from murine stool samples

#### Sample preparation

Fresh stool samples were collected directly from mice and snap frozen. gDNA was isolated using a QIAamp DNA Stool Mini Kit (QIAGEN, 51504) according to the manufacturer’s protocols.

#### Library preparation and sequencing

Libraries were prepared using the NEBNext Ultra DNA Library Prep Kit for Illumina (E7370L) according to the manufacturer’s protocol. Briefly, 50 ng of DNA was fragmented to approximately 400 bp and subjected to end repair plus ‘A’-tailing, ligation of NEB adaptor and Uracil excision by USER enzyme. Then, adaptor-ligated DNA was amplified for eight cycles by PCR using indexed primers. All purification steps were performed using AMPure XP Beads (A63881). Final libraries were analysed using an Agilent DNA 1000 chip to estimate the quantity and check size distribution, and were then quantified by qPCR using the KAPA Library Quantification Kit (KK4835, KapaBiosystems) before amplification with Illumina’s cBot. Libraries were sequenced (2 × 125 bp) on Illumina’s HiSeq 2500.

#### Taxonomic analysis

Reads were aligned to the mm10 genome using STAR 2.7.0a with default parameters^80^. DNA contaminated reads were filtered out from the analysis. The first and final ten bases of the non-contaminated reads were trimmed using DADA2 1.10.1 (ref. ^81^). Taxonomic assignments were carried out through Kaiju 1.7.0 (ref. ^82^) using the microbial subset of the NCBI BLAST non-redundant protein database (nr). Resulting sequencing counts were aggregated at genus level. Reads that could not be assigned to any specific genus were classified to the nearest known taxonomic rank (marked by the term _un). The gut microbial compositional plot displays the relative abundances (percentage) at genus level. Only the 17 most abundant taxa are shown, while the rest were moved to the ‘others’ category. For all genera, the treatment effect (finish versus start) was compared between OSKM and control (WT) mice. This was accounted in a model with an interaction term (drug:treatment) using DESeq2 with default options^83^. The paired nature of the experimental design was taken into account in the model as an adjusting factor.

#### Functional annotations

Decontamination from host and trimming was done following the same routines as for the taxonomic analysis. Cleaned sequences for all samples were assembled into contigs using megahit 1.2.4 (ref. ^84^), and prodigal 2.6.3 (ref. ^85^) was then used to predict the open reading frames inside the obtained contigs. Protein mapping and KEGG and COG annotations were obtained using the EggNOG mapper 2.0.0 (ref. ^86^). The abundance of the annotated genes was finally measured by counting aligned reads to them via Bowtie2, version 2.2.2, under default parameters^87^. Resulting counts data were aggregated at protein level. The treatment effect (finish versus start) was compared between OSKM and control (WT) mice. This was accounted in a model with an interaction term (drug:treatment) using DESeq2 with default options^83^. The paired nature of the experimental design was considered in the model as an adjusting factor. The top 500 protein hits from the fitted model (nondirectional set) as well as the top 200 positive hits and the top 200 negative hits (directional sets), in all cases ordered by statistical significance, were used to explore enrichment of functional annotations. In this regard, GO terms for bacteria and archaea were considered using the AmiGO 2 GO annotations database^88^, removing from the analysis gene sets with few genes (less than 8) and too many genes (more than 499). Statistically enriched GO terms were identified using the standard hypergeometric test. Significance was defined by the adjusted *P* value using the Benjamini and Hochberg multiple-testing correction. To take into consideration the compositional nature of the data, all DESeq2-based results were complemented with graphical representations of abundance log-ratio (between finish and start matched samples) rankings. This provides a scale invariant way (with regard to the total microbial load) to present the data^89^.

### Metabolomic analysis

#### Mouse serum

Blood was collected via submandibular vein bleed (D0, D2, D4) or intracardiac puncture following deep carbon dioxide anaesthetisation (D7) at approximately 12:00–14:00 h (4–6 h into the light cycle) of each day. Whole blood was spun down for 10 min at 3,381*g* at 4 °C and supernatant (serum) was separated and stored at −80 °C.

### In vivo time-course metabolomics analysis

#### Standard and reagents

Acetonitrile (Sigma-Aldrich), isopropanol (Sigma-Aldrich), methanol (Sigma-Aldrich), chloroform (Sigma-Aldrich), acetic acid (Sigma-Aldrich), formic acid (Sigma-Aldrich), methoxyamine hydrochloride (Sigma-Aldrich), MSTFA (*N*-methyl-*N*-(trimethylsilyl) trifluoroacetamide; Sigma-Aldrich), pyridine (Sigma-Aldrich), 3-nitrophenylhydrazine (Sigma-Aldrich), *N*-(3-dimethylaminopropyl)-*N*′-ethylcarbodiimide hydrochloride (EDC; Sigma-Aldrich) and sulfosalicylic acid (Sigma-Aldrich) as previously described^90^.

#### Sample preparation serum (lithium heparin)

A volume of 25 µl of serum were mixed with 250 µl a cold solvent mixture with ISTD (methanol/water/chloroform, 9:1:1, −20 °C), into 1.5 ml microtube, vortexed and centrifugated (10 min at 15,000*g*, 4 °C). The upper phase of supernatant was split into three parts: 50 µl was used for gas chromatography coupled to mass spectrometry (GC–MS) experiments in the injection vial, 30 µl was used for the short-chain fatty acid ultra-high performance liquid chromatography (UHPLC)–MS method, and 50 µl was used for other UHPLC–MS experiments.

#### Widely targeted analysis of intracellular metabolites GC coupled to a triple-quadrupole mass spectrometer

The GC–MS/MS method was performed on a 7890B gas chromatography system (Agilent Technologies) coupled to a triple-quadrupole 7000C (Agilent Technologies) equipped with a high-sensitivity electronic impact source (EI) operating in positive mode.

#### Targeted analysis of bile acids by ion pairing UHPLC coupled to a triple-quadrupole mass spectrometer

Targeted analysis was performed on an RRLC 1260 system (Agilent Technologies) coupled to a triple-quadrupole 6410 (Agilent Technologies) equipped with an electrospray source operating in positive mode. Gas temperature was set to 325 °C with a gas flow of 12 l min^−1^. Capillary voltage was set to 4.5 kV.

#### Targeted analysis of polyamines by ion pairing UHPLC coupled to a triple-quadrupole mass spectrometer

Targeted analysis was performed on an RRLC 1260 system (Agilent Technologies) coupled to a triple-quadrupole 6410 (Agilent Technologies) equipped with an electrospray source operating in positive mode. The gas temperature was set to 350 °C with a gas flow of 12 l min^−1^. The capillary voltage was set to 3.5 kV.

#### Targeted analysis of short-chain fatty acid by ion pairing UHPLC coupled to a 6500 + QTRAP mass spectrometer

Targeted analysis was performed on an RRLC 1260 system (Agilent Technologies) coupled to a 6500 + QTRAP (Sciex) equipped with an electrospray ion source.

#### Untargeted analysis of intracellular metabolites by UHPLC coupled to a Q-Exactive mass spectrometer (reversed-phase acetonitrile method)

The profiling experiment was performed with a Dionex Ultimate 3000 UHPLC system (Thermo Scientific) coupled to a Q-Exactive (Thermo Scientific) equipped with an electrospray source operating in both positive and negative mode and full scan mode from 100 to 1,200 *m/z*. The Q-Exactive parameters were: sheath gas flow rate, 55 arbitrary units (a.u.); auxiliary gas flow rate, 15 a.u.; spray voltage, 3.3 kV; capillary temperature, 300 °C; S-Lens RF level, 55 V. The mass spectrometer was calibrated with sodium acetate solution dedicated to low mass calibration.

#### MetaboAnalyst

The peak areas (corrected to quality control) corresponding to each annotated metabolite identified in the serum of reprogrammable mice (*n* = 6 per group) at day 5 and day 7 after doxycycline treatment were converted to log_2_ values. Data were represented as log_2_ fold change (log_2_ FC) values to each mouse at day 0 (before doxycycline administration). Metabolic pathway impact was calculated by Global ANOVA pathway enrichment and Out-degree Centrality Topology analysis through the MetaboAnalyst 4.0 software^91^, using KEGG library (2019) as a reference. The colour gradient from white to red indicates the *P* value, where red is most significant. Bubble size indicates the relative contribution of the detected metabolites in their respective KEGG pathway. Pathway impact scores the centrality of the detected metabolites in the pathway.

#### Doxycycline serum analysis

A total of 30 µl of mouse plasma was acidified with 3 µl solution of 15% phosphoric acid (vol/vol). Afterwards, 42 µl of methyl tert-butyl ether was added and vigorously mixed using a vortex. After 20 min of reequilibration, samples were centrifuged for 10 min at 21,130*g* at 4 °C. Next, 90 µl of acetonitrile were added to 10 µl of the aqueous phase to facilitate protein precipitation. After another cycle of centrifugation, the supernatant was transferred into a vial before LC–MS analysis.

The extracts were analysed by a UHPLC system coupled to a 6490 triple-quadrupole mass spectrometer (QqQ, Agilent Technologies) with electrospray ion source (LC–ESI–QqQ) working in positive mode. The injection volume was 3 µl. An ACQUITY UPLC BEH HILIC column (1.7 µm, 2.1 × 150 mm, Waters) and a gradient mobile phase consisting of water with 50 mM ammonium acetate (phase A) and acetonitrile (phase B) were used for chromatographic separation. The gradient was as follows: isocratic for 2 min at 98% B, from 2 to 9 min decreased to 50% B, for 30 s raised to 98%, and finally column equilibrated at 98% B until 13 min. The flow rate was 0.4 ml min^−1^. The mass spectrometer parameters were as follows: drying and sheath gas temperatures, 270 °C and 400 °C, respectively; source and sheath gas flow rates, 15 and 11 l min^−1^, respectively; nebulizer flow, 35 psi; capillary voltage, 3,000 V; nozzle voltage, 1,000 V; and iFunnel HRF and LRF, 130 and 100 V, respectively. The QqQ worked in MRM mode using defined transitions. The transitions for doxycycline and the collision energy (CE(V)) were 445 → 428(17), 445 → 98(60).

### Determination of methionine, SAM, SAH and homocysteine in serum

#### Sample preparation

In total, 25 μl of serum was mixed with 25 μl of TCEP and 70 μl of 1% formic acid in methanol. Samples were vortexed and left at −20 °C for 1 h, centrifuged for 10 min at 21,130*g* and 4 °C and transferred to glass vials for their analysis by LC–MS.

#### LC–MS analysis

LC–MS was performed with a Thermo Scientific Vanquish Horizon UHPLC system interfaced with a Thermo Scientific Orbitrap ID-X Tribrid Mass Spectrometer.

Metabolites were separated by HILIC chromatography with an InfinityLab Poroshell 120 HILIC-Z 2.7 μm, 2.1 mm × 100 mm column (Agilent Technologies). The mobile phase A was 50 mM ammonium acetate in water, and mobile phase B was acetonitrile. Separation was conducted under the following gradient: 0–2 min, isocratic 90% B; 2–6 min raised to 50% B; 6–7 min, isocratic 50% B; 7–7.2 min, increased to 90% B; 7.2–10.5 min, reequilibration column 90% B. The flow rate was 0.4 ml min^−1^. The injection volume was 5 μl.

Samples were analysed in positive mode in targeted SIM mode and the following setting: isolation window (*m/z*), 4; spray voltage, 3,500 V; sheath gas, 50 a.u.; auxiliary gas, 10 a.u.; ion transfer tube temperature, 300 °C; vaporizer temperature, 300 °C; Orbitrap resolution, 120,000; RF lens, 60%; AGC target, 2e5; maximum injection time, 200 ms.

SAM (*m/z* 399.145) was monitored from 5–7 min; Met (*m/z* 150.0583) from 3.2–5.2 min; SAH (*m/z* 385.1289) from 4–6 min; Hcy (*m/z* 136.0428) from 3.4–5.5 min, as previously optimized using pure standards.

### Determination of cyanocobalamin in stool

#### Sample preparation

Approximately, 20 mg of dry and pulverized stool samples were mixed with with 75 μl of TCEP and 210 μl of 1% formic acid in methanol. Samples were vortexed and subjected to three freeze–thaw cycles using liquid nitrogen. Subsequently, samples were left in ice for 1 h, centrifuged for 10 min at 21,130*g* and 4 °C and transferred to glass vials for their analysis by LC–MS.

#### LC–MS analysis

LC–MS was performed with a Thermo Scientific Vanquish Horizon UHPLC system interfaced with a Thermo Scientific Orbitrap ID-X Tribrid Mass Spectrometer.

Metabolites were separated by HILIC chromatography with an InfinityLab Poroshell 120 HILIC-Z 2.7 μm, 2.1 mm × 100 mm column (Agilent Technologies). The mobile phase A was 50 mM ammonium acetate in water, and mobile phase B was acetonitrile. Separation was conducted under the following gradient: 0–2 min, isocratic 90% B; 2–6 min raised to 50% B; 6–7 min, isocratic 50% B; 7–7.2 min, increased to 90% B; 7.2–10.5 min, reequilibration column 90% B. The flow was 0.4 ml min^−1^. The injection volume was 5 μl.

Samples were analysed in positive mode in targeted SIM mode and the following setting: isolation window (*m/z*), 4; spray voltage, 3,500 V; sheath gas, 50 a.u.; auxiliary gas, 10 a.u.; ion transfer tube temperature, 300 °C; vaporizer temperature, 300 °C; Orbitrap resolution, 120,000; RF lens, 60%; AGC target, 2e5; maximum injection time, 200 ms. Cyanocobalamin was monitored from (*m/z* 1355.5747 and *m/z* 678.291) from 5–5.5 min, as previously optimized using a pure standard.

#### Vitamin B_12_ serum analysis

Mouse serum was diluted at a 1:20 ratio in PBS and holotranscobalamin (holoTC) was measured using an ADVIA Centuar Immunoassay System (SIEMENS) with ADVIA Centuar Vitamin B_12_ Test Packs (07847260) according to the manufacturer’s instructions.

### In vitro SIL experiment

Cell pellets were mixed with 50 μl of TCEP and 140 μl of 1% formic acid in methanol (containing 150 μg l^−1^ of Tryptophan-d5 as internal standard). Samples were vortexed and subjected to three freeze–thaw cycles using liquid nitrogen. Subsequently, samples were left at −20 °C for 1 h, centrifuged for 10 min at 21,130*g* and 4 °C and transferred to glass vials for their analysis by LC–MS/MS.

#### LC–MS/MS analysis

Samples were analysed with an UHPLC 1290 Infinity II Series coupled to a QqQ/MS 6490 Series from Agilent Technologies (Agilent Technologies). The source parameters applied operating in positive electrospray ionization (ESI) were gas temperature: 270 °C; gas flow: 15 l min^−1^; nebulizer: 35 psi; sheath gas heater, 400 a.u.; sheath gas flow, 11 a.u.; capillary, 3,000 V; nozzle voltage: 1,000 V.

The chromatographic separation was performed with an InfinityLab Poroshell 120 HILIC-Z 2.7 μm, 2.1 mm × 100 mm column (Agilent Technologies), starting with 90% B for 2 min, 50% B from minute 2 to 6, and 90% B from minute 7 to 7.2. Mobile phase A was 50 mM ammonium acetate in water, and mobile phase B was acetonitrile. The column temperature was set at 25 °C and the injection volume was 2 μl.

MRM transitions for SAM (RT: 6.1 min) were 399→298 (4 V), 399→250 (12 V), 399→97 (32 V) and 399→136 (24 V) for M + 0, and 400→299 (4 V), 400→251 (12 V), 400→97 (32 V), 400→137 (24 V), 400→250 (12 V) and 400→136 (24 V) for M + 1.

### Histology

Samples were fixed overnight at 4 °C with neutral buffered formalin (HT501128-4L, Sigma-Aldrich). Paraffin-embedded tissue sections (2–3 μm in thickness) were air-dried and further dried at 60 °C overnight for immunohistochemical staining.

#### Histopathological evaluation

Sections were stained with haematoxylin and eosin (H&E) for histological evaluation by a board-certified pathologist who was blinded to the experimental groups. Additionally, periodic acid–Schiff staining (AR16592-2, Artisan, Dako, Agilent) was used to visualize mucus-producing cells on 3–4-µm sections of colon that were counterstained with haematoxylin.

In the reprogramming model, the findings were evaluated by focusing mainly on the appearance of hyperplastic and dysplastic changes of the epithelial cells of the digestive mucosa and pancreatic acini. Inflammation and loss of the intestinal goblet cells were also reported. To document the severity and extension, a semi-quantitative grading system was used based on previously used histological criteria:

- Gastric and colon mucosa inflammatory cell infiltrate and multifocal areas of crypt (large intestine) or glandular (stomach) epithelial cell dysplasia were scored from 0 to 5, where 0 indicates absence of lesion and 5 indicates very intense lesions.
- Intestinal crypt hyperplasia: 1, slight; 2, twofold to threefold increase of the crypt length; 3, >threefold increase of the crypt length.
- Goblet cell loss of the mucosa of the large intestine: 1, <10% loss; 2, 10–50% loss; 3, >50% loss.
- Histological total score was presented as a sum of all parameters scored for a given tissue.

In the colitis model, the following parameters were semi-quantitatively evaluated as previously described^92^ as follows:

- Inflammation of the colon mucosa: 0, none; 1, slight, 2, moderate; 3, severe.
- Depth of the injury: 0, none; 1, mucosa; 2, mucosa and submucosa; 3, transmural.
- Crypt damage: 0, none; 1, basal and 1/3 damaged; 2, basal and 2/3 damaged; 3, only the surface epithelium intact; 4, entire crypt and epithelium lost.
- Tissue involvement: 0, none; 1, 0–25%; 2, 26–50%; 3, 51–75%; 4, 76–100%.

The score of each parameter was multiplied by the factor of tissue involvement and summed to obtain the total histological score.

### Immunohistochemistry

Immunohistochemistry was performed using a Ventana discovery XT for NANOG and Sca1/Ly6A/E, the Leica BOND RX Research Advanced Staining System for H3K36me3, keratin 14 and vitamin B_12_, and manually for Ki67. Antigen retrieval for NANOG was performed with Cell Conditioning 1 buffer (950-124, Roche) and for Sca1/Ly6A/E with Protease 1 (5266688001, Roche) for 8 min followed with the OmniMap anti-Rat HRP (760-4457, Roche) or OmniMap anti-Rb HRP (760-4311, Roche). Blocking was done with casein (760-219, Roche). Antigen–antibody complexes were revealed with ChromoMap DAB Kit (760-159, Roche). For H3K36me3 and keratin 14, antigen retrieval was performed with BOND Epitope Retrieval 1 (AR9961, Leica) and for vit B_12_ with BOND Epitope Retrieval Solution 2 (Leica Biosystems, AR9640) for 20 min, whereas for Ki67, sections were dewaxed as part of the antigen retrieval process using the low pH EnVision FLEX Target Retrieval Solutions (Dako) for 20 min at 97 °C using a PT Link (Dako-Agilent). Blocking was performed with Peroxidase-Blocking Solution at room temperature (RT; S2023, Dako-Agilent) and 5% goat normal serum (16210064, Life technology) mixed with 2.5% BSA diluted in wash buffer for 10 and 60 min at RT. Vitamin B_12_ also was blocked with Vector M.O.M. Blocking Reagent (MK-2213, Vector) following the manufacturer’s procedures for 60 min. Primary antibodies were incubated for 30, 60 or 120 min. The secondary antibody used was the BrightVision poly HRP-Anti-Rabbit IgG, incubated for 45 min (DPVR-110HRP, ImmunoLogic) or the polyclonal goat Anti-Mouse at a dilution of 1:100 for 30 min (Dako-Agilent, P0447). Antigen–antibody complexes were revealed with 3-3′-diaminobenzidine (K346811, Agilent or RE7230-CE, Leica). Sections were counterstained with haematoxylin (CS700, Dako-Agilent or RE7107-CE, Leica) and mounted with Mounting Medium, Toluene-Free (CS705, Dako-Agilent) using a Dako CoverStainer. Specificity of staining was confirmed by staining with a rat IgG (6-001-F, R&D Systems, Bio-Techne), a Rabbit IgG (ab27478, Abcam) or a mouse IgG1, kappa (Abcam, ab18443) isotype controls. See Supplementary Table 5 for primary antibody details.

#### In situ hybridization—RNAscope

Ready-to-use reagents from RNAscope 2.5 LS Reagent Kit-RED (322150, RNAScope, ACD Bio-Techne) were loaded onto the Leica Biosystems BOND RX Research Advanced Staining System according to the user manual (322100-USM). FFPE tissue sections were baked and deparaffinized on the instrument, followed by epitope retrieval (using Leica Epitope Retrieval Buffer 2 at 95 °C for 15 min) and protease treatment (15 min at 40 °C). Probe hybridization, signal amplification, colorimetric detection and counterstaining were subsequently performed following the manufacturer’s recommendations.

Hybridization was performed with the RNAscope LS 2.5 Probe - Mm-Lgr5 - *Mus musculus* leucine rich repeat containing G-protein-coupled receptor 5 (312178, RNAScope, ACD Bio-Techne). Control probe used was the RNAscope 2.5 LS Probe - Mm-UBC - *Mus musculus* ubiquitin C (Ubc), as a housekeeping gene (310778, RNAScope - ACD Bio-Techne). The bacterial probe RNAscope 2.5 LS Negative Control Probe_dapB was used as a negative control (312038, RNAScope - ACD Bio-Techne).

#### Image acquisition

Brightfield images were acquired with a NanoZoomer-2.0 HT C9600 digital scanner (Hamamatsu) equipped with a ×20 objective. All images were visualized with a gamma correction set at 1.8 in the image control panel of the NDP.view 2 U12388-01 software (Hamamatsu, Photonics).

#### Image analysis

Brightfield images of immunohistochemistry were quantified using QuPath software^93^ with standard detection methods. Where the percentage of tissue staining is calculated, pixels were classified as positive and negative using the Thresholder function. Where the percentage of cells is quantified, the Positive Cell Detection function was used.

### Cellular and molecular methods

#### Cell culture

MEFs were cultured in standard DMEM medium with 10% FBS (Gibco, LifeTechnologies, 10270106) with antibiotics (100 U ml^−1^ penicillin–streptomycin; Life Technologies, 11528876). Reprogramming of the doxycycline-inducible 4-Factor (i4F) MEFs with inducible expression of the four Yamanaka factors Oct4, Sox2, Klf4 and cMyc (OSKM) was performed as previously described^3^. Briefly, i4F MEFs were seeded at a density of 3 × 10^5^ cells per well in six-well tissue culture plates coated with gelatin and treated with doxycycline (PanReac, A2951) 1 mg ml^−1^ continuously to induce expression of the OSKM transcription factors in the presence of ‘complete KSR media’ (15% (vol/vol) Knockout Serum Replacement (KSR, Invitrogen, 10828028) in DMEM with GlutaMax (Life Technologies, 31966047) basal media, with 1,000 U ml^−1^ LIF (Merck, 31966047), non-essential amino acids (Life Technologies, 11140035) and 100 μM beta-mercaptoethanol (Life Technologies, 31350010) plus antibiotics (penicillin–streptomycin, Gibco, 11528876)), which was replaced every 48–72 h. After 10 d, iPS cell colonies were scored by alkaline phosphatase staining according to the manufacturer’s protocol (AP blue membrane substrate detection kit, Sigma, AB0300). Vitamin B_12_ (Sigma, V2876; 2 μM final), MAT2Ai PF-9366 (MedChemExpress, HY-107778; 2 µM final), SAM (S-(5′-adenosyl)-l-methionine iodide, Merck, A4377; 100 µM final) and NSC636819 (Sigma-Aldrich, 5.31996; 10 µM final) were added continuously to the culture media and replaced every 48–72 h.

#### Retroviral reprogramming

Reprogramming of WT MEFs was performed as previously described^94^. Briefly, HEK-293T (American Type Culture Collection, ATCC-CRL-3216) cells were cultured in DMEM supplemented with 10% FBS and antibiotics (penicillin–streptomycin, Gibco, 11528876). Around 5 × 10^6^ cells per 100-mm-diameter dish were transfected with the ecotropic packaging plasmid pCL-Eco (4 μg) together with one of the following retroviral constructs (4 μg): pMXs-Klf4, pMXs-Sox2, pMXs-Oct4 or pMXs-cMyc (obtained from Addgene) using Fugene-6 transfection reagent (Roche) according to the manufacturer’s protocol. The following day, media were changed and recipient WT MEFs to be reprogrammed were seeded (1.5 × 10^5^ cells per well of a six-well plate). Retroviral supernatants (10 ml per plate/factor) were collected serially during the subsequent 48 h, at 12-h intervals, each time adding fresh media to the 293T cells cells (10 ml). After each collection, supernatant was filtered through a 0.45-µm filter, and each well of MEFs received 0.5 ml of each of the corresponding retroviral supernatants (amounting to 2 ml total). Vitamin B_12_ supplementation (Sigma, V2876; 2 µM final concertation) began on the same day as viral transduction. This procedure was repeated every 12 h for 2 d (a total of four additions). After infection was completed, media were replaced by ‘complete KSR media’ (see above). Cell pellets were harvested on day 5 (relative to the first infection) and histone extracts were processed for immunoblot as described below. On day 14 (relative to the first infection), iPS cell colonies were scored by alkaline phosphatase staining according to the manufacturer’s protocol (AP blue membrane substrate detection kit; Sigma, AB0300).

#### SIL

Doxycycline-inducible i4F MEFs were cultured as described in ‘Cell culture’ above, with 1 mg ml^−1^ doxycycline, with without continuous vitamin B_12_ supplementation. At 72 h after the addition of doxycycline, cells were transferred to complete KSR media containing a final concentration of 0.5 mM l-Serine-^13^C_3_ (Sigma-Aldrich, 604887). This is the same concentration of unlabelled l-serine normally found in the complete KSR media, and was generated by ordering custom, serine-free DMEM (Life Technologies, ME22803L1) and custom, serine-free non-essential amino acid mixture (Life Technologies, ME22804L1). Six hours after the addition of labelled media, a subset of wells was harvested by scraping in PBS and centrifugation (300*g* for 5 min); supernatant was removed and pellets were snap frozen. At 72 h after the addition of the labelled media (that is, 6 days into reprogramming), cells still in culture were transferred back to unlabelled complete KSR media, which was changed every 48–72 h. iPS cell colonies were analysed by alkaline phosphatase staining according to the manufacturer’s protocol (AP blue membrane substrate detection kit; Sigma, AB0300) on day 10. Doxycycline and vitamin B_12_ supplementation were continuous throughout the entire reprogramming protocol, and replenished with every media change (that is, every 48–72 h).

#### Histone array

i4F MEFs were cultured in the presence doxycycline ±2 µM of vitamin B_12_ over 3 or 10 days (culture conditions as described above) and histone extracts were prepared using EpiQuik Total Histone Extraction Kit (EpiGentek, OP-0006-100) according to the manufacturer’s instructions. Around 200 ng of total histone extract was used per well in the EpiQuik Histone H3 Modification Multiplex Assay Kit (Colorimetric; EpiGentek, P-3100) according to the manufacturer’s instructions.

#### Cell lysis and immunoblot

Histone extracts were prepared using an EpiQuik Total Histone Extraction Kit (EpiGentek, OP-0006-100) according to the manufacturer’s instructions and quantified using DC Protein Assay Kit (Bio-Rad, 5000111). Whole-cell extracts were prepared in RIPA buffer (10 mM Tris-HCl, pH 8.0; 1 mM EDTA; 0.5 mM EGTA; 1% Triton X-100; 0.1% sodium deoxycholate; 0.1% SDS; 140 mM NaCl). A total of 10 μg of lysate was loaded per lane and hybridized using antibodies against H3K36me3, MS, vinculin, total histone H3 and LI-COR fluorescent secondary reagents (IRDye 800 CW anti-mouse, 926-32210; IRDye 680 CW anti-mouse, 926-68070; IRDye 800 CW anti-rabbit, 926-32211; IRDye 680 CW anti-mouse, 926-68071) all at a dilution of 1:10,000 according to manufacturer’s instructions. Immunoblots were visualized on an Odyssey FC Imaging System (LI-COR Biosciences). See Supplementary Table 5 for primary antibody details.

#### GSEA

GSEAPreranked was used to perform a GSEA of annotations from MsigDB M13537, with standard GSEA and leading edge analysis settings. We used the RNA-seq gene list ranked by log_2_ fold change, selecting ‘gene set’ as the permutation method with 1,000 permutations for Kolmogorov–Smirnoff correction for multiple testing^95^.

#### Selection of genes to measure by qPCR from methionine deprivation signature

Genes belonging to the leading edge of the GSEA using the Met derivation signature (MsigDB, M13537) in the pancreas of reprogramming mice were selected. These genes were then compared to genes belonging to the leading edge of the same gene signature from i4F MEFs treated with doxycycline in vitro for 72 h, as compared to OSKM MEFs treated with vitamin B_12_ (that is, genes in MsigDB M13537 whose upregulation was relieved by B_12_ supplementation in vitro). We selected 11 of these genes for which we had qPCR primers available.

#### Analysis of mRNA levels by qPCR

Total RNA was extracted from MEFs with TRIzol (Invitrogen) according to the manufacturer’s instructions. Up to 5 µg of total RNA was reverse transcribed into cDNA using the iScript Advanced cDNA Synthesis Kit (Bio-Rad, 172-5038; pancreas) or iScript cDNA Synthesis Kit (Bio-Rad, 1708890; all other organs) for RT–qPCR. Real-time qPCR was performed using GoTaq qPCR Master Mix (Promega, A6002) in a QuantStudio 6 Flex thermocycler (Applied Biosystem) or 7900HT Fast Real-Time PCR System (Thermo Fisher). See Supplementary Table 6 for primer sequences.

#### ChIP sample preparation

i4F MEFs were cultured in the presence or absence of doxycycline ±2 µM of vitamin B_12_ (Merck, V2876) over 3 days in six-well plates (culture conditions as described above). Cells were fixed with 1% (vol/vol) PFA (Fisher Scientific, 50980487) for 2 min and then quenched with 750 mM Tris (PanReac AppliChem, A2264) for 5 min. Cells were washed twice with PBS, scraped, and spun down at 1,200*g* for 5 min. Pellets were lysed with 100 µl (per well) lysis buffer (50 mM HEPES-KOH pH 7.5, 140 mM HCl, 1 mM EDTA pH 8, 1% Triton X-100, 0.1% sodium deoxycholate, 0.1% SDS, protease inhibitor cocktail; Sigma, 4693159001) on ice for 10 min, then sonicated using a Diagenode BioRuptor Pico (Diagenode, B01060010) for ten cycles (30 s on, 30 s off) at 4 °C. Lysates were clarified for 10 min at 8,000*g*, 1% input samples were reserved, and supernatant was used for immunoprecipitation with Diagenode Protein A-coated Magnetic beads ChIP–seq grade (Diagenode, C03010020-660) and H3K3me3 monoclonal antibody (Cell Signaling Technologies, 4909) with 0.1% BSA (Sigma, 10735094001). The following day, cells were washed once with each buffer: low salt (0.1% SDS, 1% Triton X-100, 2 mM EDTA, 20 mM Tris-HCl pH 8.0, 150 mM NaCl), high salt (0.1% SDS, 1% Triton X-100, 2 mM EDTA, 20 mM Tris-HCl pH 8.0, 5,000 mM NaCl), LiCl (0.25 M LiCl, 1% NP-40, 1% sodium deoxycholate, 1 mM EDTA, 10 mM Tris-HCl pH 8.0) and eluted in 1% SDS, 100 mM NaHCO_3_ buffer. Cross-links were reversed with RNase A (Thermo Fisher, EN0531), proteinase K (Merck, 3115879001) and sodium chloride (Sigma, 71376), and chromatin fragments were purified using QIAquick PCR purification kit (Qiagen, 28104).

#### RNA-seq RNA extraction

i4F MEFs were cultured in the presence or absence of doxycycline and the indicated compounds over 3 days in six-well plates (culture conditions as described above). After 72 h, RNA was extracted using an RNeasy Kit (Qiagen, QIA74106) according to the manufacturer’s instructions.

#### ChIP–seq

The concentration of the DNA samples (inputs and immunoprecipitations) was quantified with a Qubit dsDNA HS kit, and fragment size distribution was assessed with the Bioanalyzer 2100 DNA HS assay (Agilent). Libraries for ChIP–seq were prepared at the IRB Barcelona Functional Genomics Core Facility. Briefly, single-indexed DNA libraries were generated from 0.5–1.5 ng of DNA samples using the NEBNext Ultra II DNA Library Prep kit for Illumina (New England Biolabs). Eleven cycles of PCR amplification were applied to all libraries.

The final libraries were quantified using the Qubit dsDNA HS assay (Invitrogen) and quality controlled with the Bioanalyzer 2100 DNA HS assay (Agilent). An equimolar pool was prepared with the 24 libraries and sequenced on a NextSeq 550 (Illumina). 78.9 Gb of SE75 reads were produced from two high-output runs. A minimum of 23.97 million reads were obtained for all samples.

#### RNA-seq

##### For MEFs 1–3

The concentration of total RNA extractions was quantified with the Nanodrop One (Thermo Fisher), and RNA integrity was assessed with the Bioanalyzer 2100 RNA Nano assay (Agilent). Libraries for RNA-seq were prepared at the IRB Barcelona Functional Genomics Core Facility. Briefly, mRNA was isolated from 1.5 μg of total RNA using the kit NEBNext Poly(A) mRNA Magnetic Isolation Module (New England Biolabs). The isolated mRNA was used to generate dual-indexed cDNA libraries using the NEBNext Ultra II Directional RNA Library Prep Kit for Illumina (New England Biolabs). Ten cycles of PCR amplification were applied to all libraries.

The final libraries were quantified using the Qubit dsDNA HS assay (Invitrogen) and quality controlled with the Bioanalyzer 2100 DNA HS assay (Agilent). An equimolar pool was prepared with the 12 libraries and submitted for sequencing at the Centre Nacional d’Anàlisi Genòmica (CRG-CNAG). A final quality control by qPCR was performed by the sequencing provider before paired-end 50-nucleotide sequencing on a NovaSeq 6000 S2 (Illumina). Around 77.7 Gb of PE50 reads were produced from three NovaSeq 6000 flow cells. A minimum of 55.7 million reads were obtained for all samples (Extended Data Fig. 7).

##### For MEFs 4–5

Total RNA extractions were quantified with a Nanodrop One (Thermo Fisher), and RNA integrity was assessed with the Bioanalyzer 2100 RNA Nano assay (Agilent). Libraries for RNA-seq were prepared at the IRB Barcelona Functional Genomics Core Facility. Briefly, mRNA was isolated from 1.2 μg of total RNA and used to generate dual-indexed cDNA libraries with the Illumina Stranded mRNA ligation kit (Illumina) and UD Indexes Set A (Illumina). Ten cycles of PCR amplification were applied to all libraries.

Sequencing-ready libraries were quantified using the Qubit dsDNA HS assay (Invitrogen) and quality controlled with the Tapestation HS D5000 assay (Agilent). An equimolar pool was prepared with the 15 libraries for SE75 sequencing on a NextSeq 550 (Illumina). Sequencing output was above 539 million 75-nucleotide single-end reads and a minimum of 28 million reads was obtained for all samples (Extended Data Fig. 7).

### Bioinformatic analysis

#### RNA-seq data processing

All analyses were performed in the R programming language (version 4.0.5)^96^ unless otherwise stated. Stranded paired-end reads were aligned to the *Mus musculus* reference genome version mm10 using STAR^80^ with default parameters. STAR indexes were built using the ENSEMBL annotation version GRC138.97. SAM files were converted to BAM and sorted using sambamba (version 0.6.7)^97^. Gene counts were obtained with the featureCounts function from the Rsubread package^98^ with the gtf file corresponding to ENSEMBL version GRC138.97 and parameters set to: isPairedEnd = TRUE and strandSpecific = 2. Technical replicates were collapsed by adding the corresponding columns in the count matrix.

#### Reprogramming score

We obtained a reprogramming gene signature from published data^48^ and selected genes with false discovery rate (FDR) lower than 0.05 and fold change between MEF and d3-EFF larger than 2. The reprogramming score was defined as the average of all genes in the signature after scaling the rlog transformed matrix.

#### Computation of cryptic transcript ratios between first and intermediate exons

Exon counts were generated using the featureCounts function with parameters: isPairedEnd = TRUE, strandSpecific = 2, GTF.featureType = exon, GTF.attrType = transcript_id, GTF.attrType.extra = gene_id, allowMultiOverlap = TRUE and useMetaFeatures = FALSE and the same GTF as for gene counts. Technical replicates were collapsed by adding the corresponding counts. For each gene, the longest annotated transcript was selected. Genes with less than four exons of RPKMs lower than exp(−2) were discarded from the analysis. Intermediate exons were defined as those from the fourth to the penultimate. A total of 9,365 genes were used to compute the ratio between the intermediate and first exons. Fold changes between untreated and B_12_-treated samples were computed as the ratio between the exon ratios.

#### Comparison of cryptic transcript ratios between conditions

Genes were separated by their expression after transcript length and library size normalization (RPKM). For each sample, we computed the median ratios for genes in each decile.

#### Analysis of CT in DSS time course

Data were accessed from GSE131032. Reads were processed and ratios computed as previously described. log_2_ ratios for all transcripts were summarized through the median by sample. Comparisons between days were performed fitting a linear model to the medians using ‘cage’ as a covariable. The function glht from the multcomp R package was used to find coefficients and *P* values.

#### Functional enrichment in genes with exon ratios affected by vitamin B_12_ treatment

To select genes most affected by the B_12_ treatment after reprogramming, we compared ratios between the doxy and MEF conditions and between the doxy and doxy + B_12_ conditions. Genes that increased the ratios in the first comparison (upper 25th percentile) and decreased the ratio in the second comparison (bottom 25%) were selected for functional enrichment analysis. A hypergeometric test was performed to find significant overlap between the defined gene set and the Biological Processes GO collection^99^.

#### ChIP–seq data processing

Reads were aligned to the mm10 reference genome with bowtie^100^ version 0.12.9 with parameters --n 2 and --m 1 to keep reads with multiple alignments in one position. SAM files were converted to BAM and sorted using sambamba version 0.6.7.

#### Heat maps of average coverage in gene bodies

For each sample, aligned reads were imported into R using the function scanBam from the Rsamtools package^101^. Whole-genome coverage was computed using the coverage function from the IRanges package^102^ and binned into 50-bp windows. Gene annotations were imported from Ensembl version GRCm38. The average coverage over gene bodies was computed using the normalizeToMatrix function from the EnrichedHeatmap package^103^ with parameters extend = 1,000, mean_mode = w0 and w = 50. Genes were filtered to coincide with those used in the exon ratio calculation from the RNA-seq data. Rows in the heat map were split by the average RNA-seq RPKM values in all samples.

#### Visualization of ChIP tracks

BAM files were transformed to TDF files using the count function from IGVtools (version 2.12.2)^104^ with parameters --z 7, --w 25 and --e 250. Visualization of TDF files was generated using IGV (version 2.9.4)^105^.

#### Analysis of human RNA-seq data

Data were accessed from GSE109142. Reads were processed and ratios computed as previously described except using the ENSEMBL GRCm38.101 human gene annotation and the hg38 genome assembly version. The log_2_ ratios for all transcripts were summarized through the median by sample. Comparison between diagnosis status was performed fitting a linear model to the medians with sex and the expression quantiles as covariables. The model was fitted using the lm R function and coefficients and *P* values with the coeff function.

### Statistics and data availability

#### Statistical analysis and figure preparation

Unless otherwise specified, data are presented as the mean ± s.d. Statistical analysis was performed by Student’s *t*-test or one-way analysis of variance (ANOVA) as indicated, using GraphPad Prism v9.0.0, and specific statistical tests as indicated for each experiment for bioinformatic analyses. *P* values of less than 0.05 were considered as statistically significant. No statistical methods were used to predetermine sample size in the mouse studies, but our sample sizes are similar to those reported in previous publications^3,9,16,17,19^. Animals and data points were not excluded from analysis with the exception of the MEFs that failed to reprogram in the ChIP experiment, which is clearly detailed in the text. Mice were allocated at random to treatment groups, with attempts to balance initial body weight and sex as possible. The investigators were blinded during histological assessment of the mice; other data collection and analysis was not performed blind to the conditions of the experiments. Data distribution was assumed to be normal, but this was not formally tested. Figures were prepared using Illustrator CC 2019 (Adobe).

### Reporting summary

Further information on research design is available in the Nature Portfolio Reporting Summary linked to this article.

### Supplementary information

Reporting Summary

Supplementary Tables 1–6 Excel file containing Supplementary Tables 1–6 as individual sheets.

### Source data

Source Data Fig. 1 Data and statistical analysis for all graphs/plots.

Source Data Fig. 2 Data and statistical analysis for all graphs/plots.

Source Data Fig. 2 Uncropped western blots.

Source Data Fig. 3 Data and statistical analysis for all graphs/plots.

Source Data Fig. 3 Uncropped western blots.

Source Data Fig. 4 Data and statistical analysis for all graphs/plots.

Source Data Fig. 5 Data and statistical analysis for all graphs/plots.

Source Data Extended Data Fig. 1 Data and statistical analysis for all graphs/plots.

Source Data Extended Data Fig. 2 Data and statistical analysis for all graphs/plots.

Source Data Extended Data Fig. 3 Data and statistical analysis for all graphs/plots.

Source Data Extended Data Fig. 4 Data and statistical analysis for all graphs/plots.

Source Data Extended Data Fig. 5 Data and statistical analysis for all graphs/plots.

Source Data Extended Data Fig. 5 Uncropped western blots.

Source Data Extended Data Fig. 6 Data and statistical analysis for all graphs/plots.

Source Data Extended Data Fig. 6 Uncropped western blots.

Source Data Extended Data Fig. 7 Data and statistical analysis for all graphs/plots.

Source Data Extended Data Fig. 8 Data and statistical analysis for all graphs/plots.

## Data Availability

All sequencing data are deposited under the following Gene Expression Omnibus accessions: GSE154149, microbial genome analysis from OSKM and WT mice pretreatment and after 7 d of 1 mg ml^−1^ treatment with doxycycline in the drinking water; GSE200578, ChIP–seq of H3k36me3 samples of OSKM reprogramming MEFs treated with B_12_; GSE200579, RNA-seq samples of OSKM reprogrammable MEFs treated with or without vitamin B_12_; GSE232382, RNA-seq samples of OSKM reprogrammable MEFs treated with vitamin B_12_ and/or various compounds to modulate SAM or histone methylation. Previously published datasets that were used for analysis in the current study are: GSE131032, RNA-seq of time-course analysis of repairing murine epithelium after DSS injury; GSE109142, RNA-seq of human paediatric ulcerative colitis and normal tissue controls; GSE102518, RNA-seq of murine in vitro reprogramming in MEFs of varying genotypes; GSE77722, RNA-seq of murine in vivo reprogramming in mice of varying genotypes. Source data are provided with this paper.

## References

[1] Takahashi K, Yamanaka S (2006). Induction of pluripotent stem cells from mouse embryonic and adult fibroblast cultures by defined factors. Cell.

[2] Taguchi J, Yamada Y (2017). In vivo reprogramming for tissue regeneration and organismal rejuvenation. Curr. Opin. Genet. Dev..

[3] Abad M (2013). Reprogramming in vivo produces teratomas and iPS cells with totipotency features. Nature.

[4] Ocampo A (2016). In vivo amelioration of age-associated hallmarks by partial reprogramming. Cell.

[5] Doeser MC, Schöler HR, Wu G (2018). Reduction of fibrosis and scar formation by partial reprogramming in vivo. Stem Cells.

[6] Lu Y (2020). Reprogramming to recover youthful epigenetic information and restore vision. Nature.

[7] Chen Y (2021). Reversible reprogramming of cardiomyocytes to a fetal state drives heart regeneration in mice. Science.

[8] Browder KC (2022). In vivo partial reprogramming alters age-associated molecular changes during physiological aging in mice. Nat. Aging.

[9] Chondronasiou D (2022). Multi-omic rejuvenation of naturally aged tissues by a single cycle of transient reprogramming. Aging Cell.

[10] Ohnishi K (2014). Premature termination of reprogramming in vivo leads to cancer development through altered epigenetic regulation. Cell.

[11] Shibata H (2018). In vivo reprogramming drives Kras-induced cancer development. Nat. Commun..

[12] Shyh-Chang N (2013). Influence of threonine metabolism on *S*-adenosylmethionine and histone methylation. Science.

[13] Fernández-Arroyo S (2016). Activation of the methylation cycle in cells reprogrammed into a stem cell-like state. Oncoscience.

[14] Degnan PH, Taga ME, Goodman AL (2014). Vitamin B12 as a modulator of gut microbial ecology. Cell Metab..

[15] Nicholson JK (2012). Host-gut microbiota metabolic interactions. Science.

[16] Mosteiro L (2016). Tissue damage and senescence provide critical signals for cellular reprogramming in vivo. Science.

[17] Melendez, E. et al. Natural killer cells act as an extrinsic barrier for in vivo reprogramming. *Development*10.1242/dev.200361 (2022). 10.1242/dev.200361 PMC9124575 35420133

[18] Kennedy EA, King KY, Baldridge MT (2018). Mouse microbiota models: comparing germ-free mice and antibiotics treatment as tools for modifying gut bacteria. Front. Physiol..

[19] Chondronasiou D (2022). Deciphering the roadmap of in vivo reprogramming toward pluripotency. Stem Cell Rep..

[20] Levy M (2015). Microbiota-modulated metabolites shape the intestinal microenvironment by regulating NLRP6 inflammasome signaling. Cell.

[21] Chang CS (2021). Identification of a gut microbiota member that ameliorates DSS-induced colitis in intestinal barrier enhanced Dusp6-deficient mice. Cell Rep..

[22] Yui S (2018). YAP/TAZ-dependent reprogramming of colonic epithelium links ECM remodeling to tissue regeneration. Cell Stem Cell.

[23] Moschen AR (2016). Lipocalin 2 protects from inflammation and tumorigenesis associated with gut microbiota alterations. Cell Host Microbe.

[24] Degnan PH, Barry NA, Mok KC, Taga ME, Goodman AL (2014). Human gut microbes use multiple transporters to distinguish vitamin B_12_ analogs and compete in the gut. Cell Host Microbe.

[25] Martens H, Barg M, Warren D, Jah J-H (2002). Microbial production of vitamin B 12. Appl. Microbiol. Biotechnol..

[26] Green R (2017). Vitamin B_12_ deficiency. Nat. Rev. Dis. Prim..

[27] Kornerup LS (2018). Tissue distribution of oral vitamin B_12_ is influenced by B_12_ status and B_12_ form: an experimental study in rats. Eur. J. Nutr..

[28] Shin O-H (1997). Methyl-group donors cannot prevent apoptotic death of rat hepatocytes induced by choline-deficiency. J. Cell. Biochem..

[29] Birn H (2002). Megalin is essential for renal proximal tubule reabsorption and accumulation of transcobalamin-B_12_. Am. J. Physiol. Ren. Physiol..

[30] Scott JSD, Treston AM, Bowman EPW, Owens JA, Cooksley WG (1984). The regulatory roles of liver and kidney in cobalamin (vitamin B_12_) metabolism in the rat: the uptake and intracellular binding of cobalamin and the activity of the cobalamin-dependent enzymes in response to varying cobalamin supply. Clin. Science.

[31] Okuda K (1962). Relationship between intake of vitamin B_12_ and its storage by the kidney in the rat. J. Nutr..

[32] Rossi M, Amaretti A, Raimondi S (2011). Folate production by probiotic bacteria. Nutrients.

[33] Sanderson SM, Gao X, Dai Z, Locasale JW (2019). Methionine metabolism in health and cancer: a nexus of diet and precision medicine. Nat. Rev. Cancer.

[34] Shiraki N (2014). Methionine metabolism regulates maintenance and differentiation of human pluripotent stem cells. Cell Metab..

[35] Wang J (2009). Dependence of mouse embryonic stem cells on threonine catabolism. Science.

[36] Bravo AC (2022). Analysis of *S*-adenosylmethionine and *S*-adenosylhomocysteine: method optimisation and profiling in healthy adults upon short-term dietary intervention. Metabolites.

[37] Lildballe DL, Mutti E, Birn H, Nexo E (2012). Maximal load of the vitamin B12 transport system: a study on mice treated for four weeks with high-dose vitamin B_12_ or cobinamide. PLoS ONE.

[38] Beedholm-Ebsen R (2010). Identification of multidrug resistance protein 1 (MRP1/ABCC1) as a molecular gate for cellular export of cobalamin. Blood.

[39] Kokkinakis DM (2006). Mitotic arrest, apoptosis, and sensitization to chemotherapy of melanomas by methionine deprivation stress. Mol. Cancer Res..

[40] Zviran A (2019). Deterministic somatic cell reprogramming involves continuous transcriptional changes governed by myc and epigenetic-driven modules. Cell Stem Cell.

[41] National Research Council (US) Subcommittee on Laboratory Animal Nutrition. *Nutrient Requirements of Laboratory Animals* (National Academies Press, 1995). 10.17226/4758 25121259

[42] Mentch SJ (2015). Histone methylation dynamics and gene regulation occur through the sensing of one-carbon metabolism. Cell Metab..

[43] Ye C, Sutter BM, Wang Y, Kuang Z, Tu BP (2017). A metabolic function for phospholipid and histone methylation. Mol. Cell.

[44] Chronis C (2017). Cooperative binding of transcription factors orchestrates reprogramming. Cell.

[45] van den Hurk M (2016). Transcriptional and epigenetic mechanisms of cellular reprogramming to induced pluripotency. Epigenomics.

[46] Chu CH (2014). KDM4B as a target for prostate cancer: structural analysis and selective inhibition by a novel inhibitor. J. Med. Chem..

[47] Edmunds JW, Mahadevan LC, Clayton AL (2008). Dynamic histone H3 methylation during gene induction: HYPB/Setd2 mediates all H3K36 trimethylation. EMBO J..

[48] Schwarz BA (2018). Prospective isolation of poised iPSC intermediates reveals principles of cellular reprogramming. Cell Stem Cell.

[49] Li B (2007). Infrequently transcribed long genes depend on the Set2/Rpd3S pathway for accurate transcription. Genes Dev..

[50] Neri F (2017). Intragenic DNA methylation prevents spurious transcription initiation. Nature.

[51] Barral A (2022). SETDB1/NSD-dependent H3K9me3/H3K36me3 dual heterochromatin maintains gene expression profiles by bookmarking poised enhancers. Mol. Cell.

[52] McCauley BS (2021). Altered chromatin states drive cryptic transcription in aging mammalian stem cells. Nat. Aging.

[53] Huang C, Zhu B (2018). Roles of H3K36-specific histone methyltransferases in transcription: antagonizing silencing and safeguarding transcription fidelity. Biophys. Rep..

[54] Pu M (2015). Trimethylation of Lys36 on H3 restricts gene expression change during aging and impacts life span. Genes Dev..

[55] Sen P (2015). H3K36 methylation promotes longevity by enhancing transcriptional fidelity. Genes Dev..

[56] Carrozza MJ (2005). Histone H3 methylation by Set2 directs deacetylation of coding regions by Rpd3S to suppress spurious intragenic transcription. Cell.

[57] Ang Y-S (2011). Wdr5 mediates self-renewal and reprogramming via the embryonic stem cell core transcriptional network. Cell.

[58] Iismaa SE (2018). Comparative regenerative mechanisms across different mammalian tissues. npj Regen. Med..

[59] Ayyaz A (2019). Single-cell transcriptomes of the regenerating intestine reveal a revival stem cell. Nature.

[60] Nusse YM (2018). Parasitic helminths induce fetal-like reversion in the intestinal stem cell niche. Nature.

[61] Perše M, Cerar A (2012). Dextran sodium sulphate colitis mouse model: traps and tricks. J. Biomed. Biotechnol..

[62] Czarnewski P (2019). Conserved transcriptomic profile between mouse and human colitis allows unsupervised patient stratification. Nat. Commun..

[63] Haberman Y (2019). Ulcerative colitis mucosal transcriptomes reveal mitochondriopathy and personalized mechanisms underlying disease severity and treatment response. Nat. Commun..

[64] Mentch SJ, Locasale JW (2016). One-carbon metabolism and epigenetics: understanding the specificity. Ann. N. Y. Acad. Sci..

[65] Boon R (2021). Metabolic fuel for epigenetic: nuclear production meets local consumption. Front. Genet..

[66] Ye C, Tu BP (2018). Sink into the epigenome: histones as repositories that influence cellular metabolism. Trends Endocrinol. Metab..

[67] Eram MS (2015). Kinetic characterization of human histone H3 lysine 36 methyltransferases, ASH1L and SETD2. Biochim. Biophys. Acta.

[68] Lurz E (2020). Vitamin B_12_ deficiency alters the gut microbiota in a murine model of colitis. Front. Nutr..

[69] Benight NM (2011). B-vitamin deficiency is protective against DSS-induced colitis in mice. Am. J. Physiol. Gastrointest. Liver Physiol..

[70] Tamura J (1999). Immunomodulation by vitamin B_12_: augmentation of CD8^+^ T lymphocytes and natural killer (NK) cell activity in vitamin B_12_-deficient patients by methyl-B_12_ treatment. Clin. Exp. Immunol..

[71] Liu M (2021). The histone methyltransferase SETD2 modulates oxidative stress to attenuate experimental colitis. Redox Biol..

[72] Ward MG (2015). Prevalence and risk factors for functional vitamin B_12_ deficiency in patients with Crohn’s disease. Inflamm. Bowel Dis..

[73] Zheng S (2017). Association of ulcerative colitis with transcobalamin II gene polymorphisms and serum homocysteine, vitamin B_12_, and folate levels in Chinese patients. Immunogenetics.

[74] Leinenkugel G (2022). Sca-1 is a marker for cell plasticity in murine pancreatic epithelial cells and induced by IFN-β in vitro. Pancreatology.

[75] Dekel B (2006). Isolation and characterization of nontubular sca-1+lin- multipotent stem/progenitor cells from adult mouse kidney. J. Am. Soc. Nephrol..

[76] Camarata TD, Weaver GC, Vasilyev A, Arnaout MA (2015). Negative regulation of TGFβ signaling by stem cell antigen-1 protects against ischemic acute kidney injury. PLoS ONE.

[77] Bender Kim CF (2005). Identification of bronchioalveolar stem cells in normal lung and lung cancer. Cell.

[78] Louie SM (2022). Progenitor potential of lung epithelial organoid cells in a transplantation model. Cell Rep..

[79] Zhang W (2013). Fluorinated *N*, *N*-dialkylaminostilbenes repress colon cancer by targeting methionine *S*-adenosyltransferase 2A. ACS Chem. Biol..

[80] Dobin A (2013). STAR: ultrafast universal RNA-seq aligner. Bioinformatics.

[81] Callahan BJ (2016). DADA2: high-resolution sample inference from Illumina amplicon data. Nat. Methods.

[82] Menzel P, Ng KL, Krogh A (2016). Fast and sensitive taxonomic classification for metagenomics with Kaiju. Nat. Commun..

[83] Love MI, Huber W, Anders S (2014). Moderated estimation of fold change and dispersion for RNA-seq data with DESeq2. Genome Biol..

[84] Li D, Liu C-M, Luo R, Sadakane K, Lam T-W (2015). MEGAHIT: an ultra-fast single-node solution for large and complex metagenomics assembly via succinct de Bruijn graph. Bioinformatics.

[85] Hyatt D (2010). Prodigal: prokaryotic gene recognition and translation initiation site identification. BMC Bioinformatics.

[86] Huerta-Cepas J (2019). EggNOG 5.0: a hierarchical, functionally and phylogenetically annotated orthology resource based on 5090 organisms and 2502 viruses. Nucleic Acids Res..

[87] Langmead B, Salzberg SL (2012). Fast gapped-read alignment with Bowtie 2. Nat. Methods.

[88] Carbon S (2009). AmiGO: online access to ontology and annotation data. Bioinformatics.

[89] Morton JT (2019). Establishing microbial composition measurement standards with reference frames. Nat. Commun..

[90] Viltard M (2019). The metabolomic signature of extreme longevity: naked mole rats versus mice. Aging.

[91] Pang Z, Chong J, Li S, Xia J (2020). MetaboAnalystR 3.0: toward an optimized workflow for global metabolomics. Metabolites.

[92] Peng X (2010). Inhibition of phosphoinositide 3-kinase ameliorates dextran sodium sulfate-induced colitis in mice. J. Pharmacol. Exp. Ther..

[93] Bankhead P (2017). QuPath: open source software for digital pathology image analysis. Sci. Rep..

[94] Li H (2009). The Ink4/Arf locus is a barrier for iPS cell reprogramming. Nature.

[95] Subramanian A (2005). Gene-set enrichment analysis: a knowledge-based approach for interpreting genome-wide expression profiles. Proc. Natl Acad. Sci. USA.

[96] R Development Core Team *R: A Language and Environment for Statistical Computing* (R Foundation for Statistical Computing, 2020).

[97] Tarasov A, Vilella AJ, Cuppen E, Nijman IJ, Prins P (2015). Sambamba: fast processing of NGS alignment formats. Bioinformatics.

[98] Liao Y, Smyth GK, Shi W (2019). The R package Rsubread is easier, faster, cheaper and better for alignment and quantification of RNA sequencing reads. Nucleic Acids Res..

[99] Ashburner M (2000). Gene Ontology: tool for the unification of biology. the Gene Ontology Consortium. Nat. Genet..

[100] Langmead B, Trapnell C, Pop M, Salzberg SL (2009). Ultrafast and memory-efficient alignment of short DNA sequences to the human genome. Genome Biol..

[101] Li H (2009). The sequence alignment/map format and SAMtools. Bioinformatics.

[102] Lawrence M (2013). Software for computing and annotating genomic ranges. PLoS Comput. Biol..

[103] Gu Z, Eils R, Schlesner M, Ishaque N (2018). EnrichedHeatmap: an R/Bioconductor package for comprehensive visualization of genomic signal associations. BMC Genomics.

[104] Thorvaldsdottir H, Robinson JT, Mesirov JP (2013). Integrative Genomics Viewer (IGV): high-performance genomics data visualization and exploration. Brief. Bioinform..

[105] Robinson JT (2011). Integrative genomics viewer. Nat. Biotechnol..

