# Supplementary material for: Vitamin B12 is a limiting factor for induced cellular plasticity and tissue repair
Source: Nat Metab. 2023 Nov 16;5(11):1911–30. doi: 10.1038/s42255-023-00916-6 (PMC10663163; doi:10.1038/s42255-023-00916-6)
Supplement: Supplementary file 1 — Reporting Summary [file 42255_2023_916_MOESM1_ESM.pdf]

nature portfolio

Corresponding author(s): Manuel Serrano

Last updated by author(s): Aug 8, 2023

Reporting Summary

Nature Portfolio wishes to improve the reproducibility of the work that we publish. This form provides structure for consistency and transparency in reporting. For further information on Nature Portfolio policies, see our Editorial Policies and the Editorial Policy Checklist.

Statistics

For all statistical analyses, confirm that the following items are present in the figure legend, table legend, main text, or Methods section.

n/a	Confirmed	
☐	☒	The exact sample size (*n*) for each experimental group/condition, given as a discrete number and unit of measurement
☐	☒	A statement on whether measurements were taken from distinct samples or whether the same sample was measured repeatedly
☐	☒	The statistical test(s) used AND whether they are one- or two-sided<br>*Only common tests should be described solely by name; describe more complex techniques in the Methods section.*
☒	☐	A description of all covariates tested
☒	☐	A description of any assumptions or corrections, such as tests of normality and adjustment for multiple comparisons
☐	☒	A full description of the statistical parameters including central tendency (e.g. means) or other basic estimates (e.g. regression coefficient) AND variation (e.g. standard deviation) or associated estimates of uncertainty (e.g. confidence intervals)
☐	☒	For null hypothesis testing, the test statistic (e.g. *F*, *t*, *r*) with confidence intervals, effect sizes, degrees of freedom and *P* value noted<br>*Give P values as exact values whenever suitable.*
☒	☐	For Bayesian analysis, information on the choice of priors and Markov chain Monte Carlo settings
☒	☐	For hierarchical and complex designs, identification of the appropriate level for tests and full reporting of outcomes
☒	☐	Estimates of effect sizes (e.g. Cohen's *d*, Pearson's *r*), indicating how they were calculated

Our web collection on statistics for biologists contains articles on many of the points above.

Software and code

Policy information about availability of computer code

Data collection	Histology: NanoZoomer-2.0 HT C9600 digital scanner (Hamamatsu)<br>bacterial sequencing: HiSeq 2500 (Illumina)<br>transcobalamin levels: ADVIA Centuar Immunoassay System (SIEMENS)<br>serum metabolomics: 7890B gas chromatography, RRLC 1260 system, 6490 triple-quadrupole mass spectrometer (Agilent Technologies), Dionex Ultimate 3000 UHPLC system, Orbitrap ID-X Tribrid Mass Spectrometer (Thermo Scientific) as specified in methods<br>immunoblots: LiCor Odyssey FC<br>qPCR: LifeTechnologies Quant Studio 6 Flex System<br>C13 stable isotope labeling metabolomics: UHPLC 1290 Infinity II Series coupled to a QqQ/MS 6490 Series from Agilent Technologies<br>ChIP-seq: NextSeq550 (Illumina)<br>RNA-seq: NovaSeq6000 S2 (Illumina) and NextSeq550 (Illumina)<br>FITC-dextran leak assay: ioTek Synergy H1 Microplate Reader
Data analysis	General statistical analyses: GraphPad Prism 9.0.0 with the statistical tests as indicated in each figure.<br>bacterial analysis: Microbiome data analyses were performed in the R programming language version 4.0.5. Reads that aligned to the mm10 genome, STAR (v2.7.0a), were filtered out. Taxonomic assignments were carried out through Kaiju (v1.7.0). Annotations were extracted from the microbial subset of the NCBI BLAST non-redundant protein database (nr). Cleaned sequences were assembled into contigs using megahit (v1.2.4). Open reading frames from the obtained contigs were predicted with prodigal (v2.6.3). Protein mapping as well as Kegg and COG annotations were obtained using the eggnog mapper (v2.0.0). Alignment to the assembled transcriptome was performed with Bowtie2 (v2.2.2). Sam files were sorted and converted to BAM format using sambamba (v0.6.7).<br>Serum metabolic pathway impact: Global ANOVA pathway enrichment and Out-degree Centrality Topology analysis through the MetaboAnalyst 4.0 software, using KEGG library (2019) as reference.

All RNASeq analyses were perfomed in the R programming language version 4.0.5. Reads were aligned to the mm10 mouse genome using STAR (v2.7.10a). Annotations were extracted from ENSEMBL (vGRC138.97).
ChIPseq data was aligned using Bowtie (v0.12.9). Sam files were sorted and converted to BAM format using sambamba (v0.6.7). IGVtools (v2.9.4) was used to generate alignment visualizations.
Histology was quantified using QuPath 0.4.3.

For manuscripts utilizing custom algorithms or software that are central to the research but not yet described in published literature, software must be made available to editors and reviewers. We strongly encourage code deposition in a community repository (e.g. GitHub). See the Nature Portfolio guidelines for submitting code & software for further information.

Data

Policy information about availability of data

All manuscripts must include a data availability statement. This statement should provide the following information, where applicable:

- Accession codes, unique identifiers, or web links for publicly available datasets
- A description of any restrictions on data availability
- For clinical datasets or third party data, please ensure that the statement adheres to our policy

All sequencing data are deposited under the following GEO accessions:
GSE154149 Microbial genome analysis from OSKM and WT mice pre-treatment and after 7 days of 1 mg/mL treatment with doxycycline in the drinking water.
GSE200578 ChIP-Seq of H3k36me3 samples of OSKM reprogramming mouse embryonic fibroblasts treated with B12
GSE200579 RNA-Seq samples of OSKM reprogrammable MEFs treated with or without vitamin B12
GSE232382 RNA-Seq samples of OSKM reprogrammable MEFs treated with vitamin B12 and/or various compounds to modulate SAM or histone methylation

Previously published datasets that were used for analysis in this study are:
GSE131032 RNA-Seq of time course of repairing murine epithelium after DSS injury
GSE109142 RNA-Seq of human pediatric ulcerative colitis and normal tissue controls
GSE102518 RNA-Seq of murine in vitro reprogramming in MEFs of varying genotypes
GSE77722 RNA-Seq of murine in vivo reprogramming in mice of varying genotypes

Human research participants

Policy information about studies involving human research participants and Sex and Gender in Research.

Reporting on sex and gender	n/a
Population characteristics	n/a
Recruitment	n/a
Ethics oversight	n/a

Note that full information on the approval of the study protocol must also be provided in the manuscript.

Field-specific reporting

Please select the one below that is the best fit for your research. If you are not sure, read the appropriate sections before making your selection.

☒ Life sciences ☐ Behavioural & social sciences ☐ Ecological, evolutionary & environmental sciences

For a reference copy of the document with all sections, see nature.com/documents/nr-reporting-summary-flat.pdf

Life sciences study design

All studies must disclose on these points even when the disclosure is negative.

Sample size	No statistical methods were used to predetermine sample size in the mouse studies. As requested by Reviewer 2, for the revision, we conducted a power analysis using the calculator published by the Chinese University of Hong Kong (http://www.lasec.cuhk.edu.hk/sample-size-calculation.html) to assess and appropriately power the analysis of the data presented in Fig. 1d
Data exclusions	In our ChIPseq (and corresponding RNAseq), we used n=3 independent MEFs. As clearly explained in the text and shown in the figures, two clones had low-to-no inherent reprogramming capacity (biologic variability) so we selected the n=1 clone with high efficiency for ChIP.
Replication	All replicate values (as indicated in Figure Legends) represent biological replicates. For qPCR experiments, biological replicates (i.e. independent mice, MEFs) were used and indicated in the n for each figure, and each sample was run in technical triplicate. For in vitro experiments, replicates were generally performed on the same day with n=2-3 cell lines as indicated, each derived from independent mouse embryos; independent experiments always included OSKM (doxy) and OSKM + B12 controls. For the fecal bacterial analysis, stool samples from biologic replicates (i.e. independent mice) were collected on the same days. For the serum metabolomic analysis, two independent

experiments were performed (i.e. untargeted metabolomics and targeted 1C metabolomics); samples for each experiment were collected on the same days/times from independent mice, and time was maintained across experiments. In vivo reprogramming experiments were done in the span of months-to-years as the project evolved; in general, independent panels represent cohorts of mice that were treated together and sacrificed together. For the DSS experiments, results represent data from pooled experiments as indicated in the figure legend, with each experiment having at least n=3 DSS and DSS + B12 control mice. All attempts at replication were successful except as indicated regarding the variability of reprogramming in the ChIP experiment.

Randomization

Mice were randomized into experimental groups based on age (i.e. always trying to maintain consistent 8-16 week age) and, when possible, initial body mass.

Blinding

All histological scoring was conducted by a blinded histopathologist.

Reporting for specific materials, systems and methods

We require information from authors about some types of materials, experimental systems and methods used in many studies. Here, indicate whether each material, system or method listed is relevant to your study. If you are not sure if a list item applies to your research, read the appropriate section before selecting a response.

Materials & experimental systems

n/a	Involved in the study
☐	☒ Antibodies
☐	☒ Eukaryotic cell lines
☒	☐ Palaeontology and archaeology
☐	☒ Animals and other organisms
☒	☐ Clinical data
☒	☐ Dual use research of concern

Methods

n/a	Involved in the study
☐	☒ ChIP-seq
☒	☐ Flow cytometry
☒	☐ MRI-based neuroimaging

Antibodies

Antibodies used

IHC: NANOG D2A3 (8822, CellSignaling), Sca1/Ly6A/E [E13 161-7] (ab51317, Abcam), H3K36me3 (Lys36) (D5A7) XP (4909, CellSignaling), Ki67 (ab15580, Abcam), Vitamin B12 (Sigma, V9505). Primary antibodies were diluted 1:100 for NANOG, 1:2000 for Ki67, 1:1000 for Ly6a/Sca1, 1:400 for H3K36me3, 1: 2000 for Keratin 14 and 1:750 for Vit B12.

ChIP: H3K36me3 (Lys36) (D5A7) XP (4909, CellSignaling)

Immunoblot: H3K36me3 (Lys36) (D5A7) XP (4909, CellSignaling) 1:1000, methionine synthase (Abcam#ab66039) 1:1000, vinculin (SCBT#sc-73614) 1:5000, total histone H3 (CellSignaling #3638) 1:1000; LI-COR fluorescent secondary reagents (IRDye 800 CW anti mouse #926-32210; IRDye 680 CW anti mouse #926-68070; IRDye 800 CW anti rabbit #926-32211; IRDye 680 CW anti mouse #926-68071) all used at 1:10 000

Validation

all IHC antibodies were validated by our histopathology facility, and IHC protocols include a secondary-only control. ChIP antibody has been validated by the manufacturer for use in mouse cells.

Eukaryotic cell lines

Policy information about cell lines and Sex and Gender in Research

Cell line source(s)

all primary mouse embryonic fibroblasts (MEFs) were generated in-house from E13.5 embryos. Cells were genotyped to determine whether they were OSKM transgenic. Cells were not sexed. HEK293T cells were purchased from the ATCC.

Authentication

all MEFs were made in-house from fresh embryos and were not authenticated further. 293T were purchased from the ATCC and come with a certificate of analysis.

Mycoplasma contamination

all MEFs were derived from embryos in-house and were used in experiments as P1; thus, no mycoplasma testing was carried out on these cells. Mycoplasma testing on all cell lines in culture is carried out once per month, and any positive cells are discarded so that no cell lines within the lab are mycoplasma positive.

Commonly misidentified lines
(See ICLAC register)

HEK293T do not appear in the ICLAC register

Animals and other research organisms

Policy information about studies involving animals; ARRIVE guidelines recommended for reporting animal research, and Sex and Gender in Research

Laboratory animals

C57/BL6 mice and OSKM mice (which are on a C57/BL6 background) aged 8-16 weeks of mixed gender were used in all experiments.

Laboratory animals	OSKM mice refer to the i4F-B strain in Abad et al Science 2013.
Wild animals	study did not involve wild animals
Reporting on sex	Sex was not considered in study design and animals of both sexes were used in all experiments, keeping as close to 50/50 as possible, as litters allowed; specific male (M) and female (F) ratios for each in vivo experiment are indicated in the figure legends next to the total n (ex. n=5; 3M 2F). We have not done sex-based analysis, as separating sex would limit the sample size.
Field-collected samples	study did not involve field collected sample
Ethics oversight	Animal experimentation at the IRB Barcelona was performed according to protocols approved by the Science Park of Barcelona (PCB) Ethics Committee for Research and Animal Welfare.

Note that full information on the approval of the study protocol must also be provided in the manuscript.

ChIP-seq

Data deposition

☒ Confirm that both raw and final processed data have been deposited in a public database such as GEO.

☒ Confirm that you have deposited or provided access to graph files (e.g. BED files) for the called peaks.

Data access links<br>*May remain private before publication.*	https://www.ncbi.nlm.nih.gov/geo/query/acc.cgi?acc=GSE200578
Files in database submission	GSM6038017_0142_2022_0071_2022_MACS2_broad_peaks_peaks.txt.gz<br>GSM6038018_0143_2022_0072_2022_MACS2_broad_peaks_peaks.txt.gz<br>GSM6038021_0144_2022_0073_2022_MACS2_broad_peaks_peaks.txt.gz<br>GSM6038022_0145_2022_0074_2022_MACS2_broad_peaks_peaks.txt.gz<br><br>RAW data has been deposited to the Sequence Read Archive (SRA) and will be made public after publication
Genome browser session<br>(e.g. UCSC)	http://genome.ucsc.edu/s/yocamille/MKovatcheva_2022

Methodology

Replicates	Only one of three MEFs was analyzed in ChIP due to poor inherent reprogramming efficiency of others, as described in the paper. Input and IP samples were generated for each condition.
Sequencing depth	reads processed 331910925<br>uniquely aligned 233201616<br>failed to align 32857274<br>multialigned 65852035<br><br>Reads were 75bps long and single end
Antibodies	H3K36me3 (D5A7) (CellSignaling #4909)
Peak calling parameters	MACS2 v. 2.2.6 was used to call peaks using parameters: callpeak --broad -g 3209286105 with INPUT sample as control
Data quality	MACS2peaks (qvalue < 0.1)<br>dox_noB12 116853<br>MEF_noB12 91216<br>MEF_B12 106819<br>dox_noB12 118386
Software	Aligner: bowtie v1.2.3 with parameters: -n 2 -p 30 -m 1 --phred33-quals<br>Sam to bam and sorting: sambamba v0.6.7<br>Peak caller: MACS2 v. 2.2.6 with parameters: callpeak --broad -g 3209286105
