# Supplementary figures and images for: Vitamin B12 is a limiting factor for induced cellular plasticity and tissue repair
Source: Nat Metab. 2023 Nov 16;5(11):1911–30. doi: 10.1038/s42255-023-00916-6 (PMC10663163; doi:10.1038/s42255-023-00916-6)

**Figure 2d Source Data:**

**Pancreas**

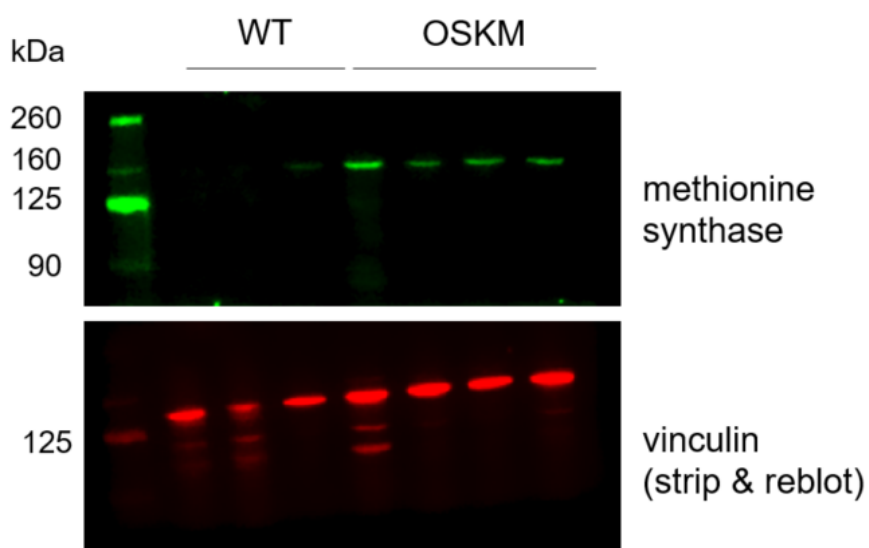

**Kidney**

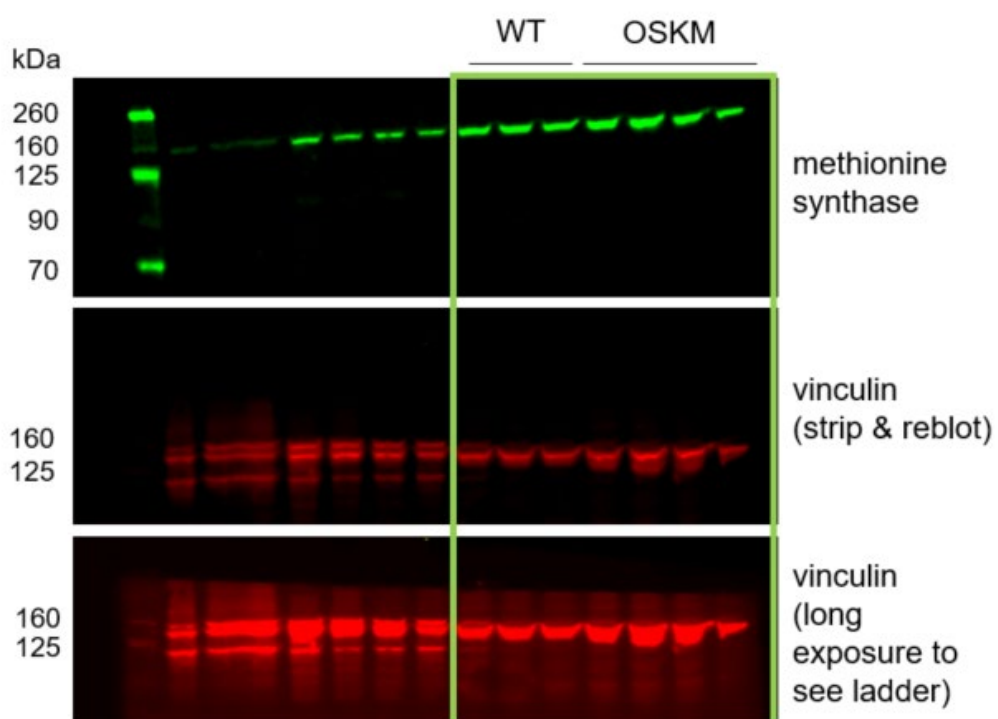

Supplement: Supplementary file 5 — Uncropped western blots. [file 42255_2023_916_MOESM5_ESM.pdf]

### Figure 3c Source Data:

Blot in paper (MEF1):

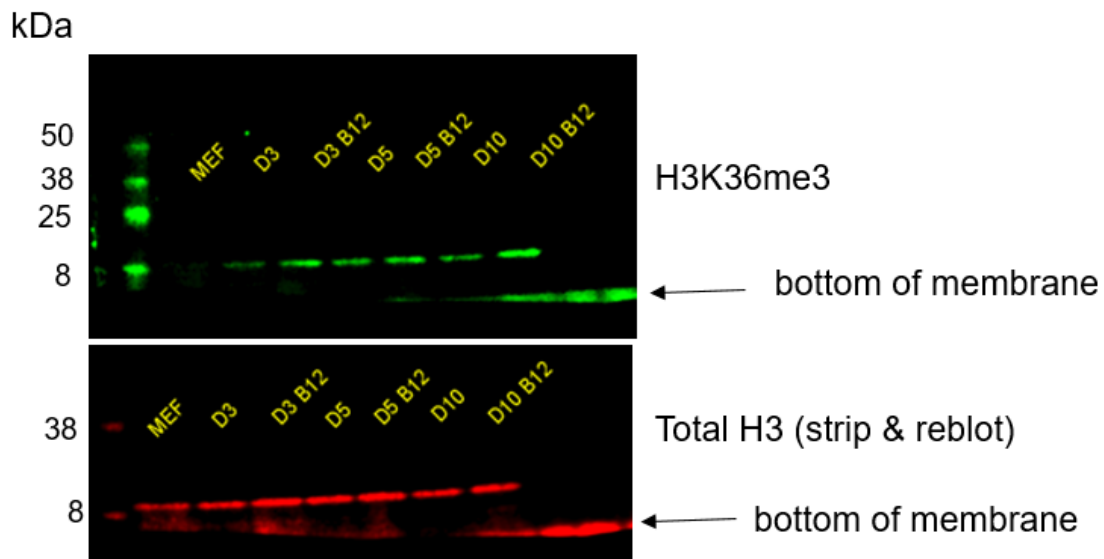

Additional MEFs:

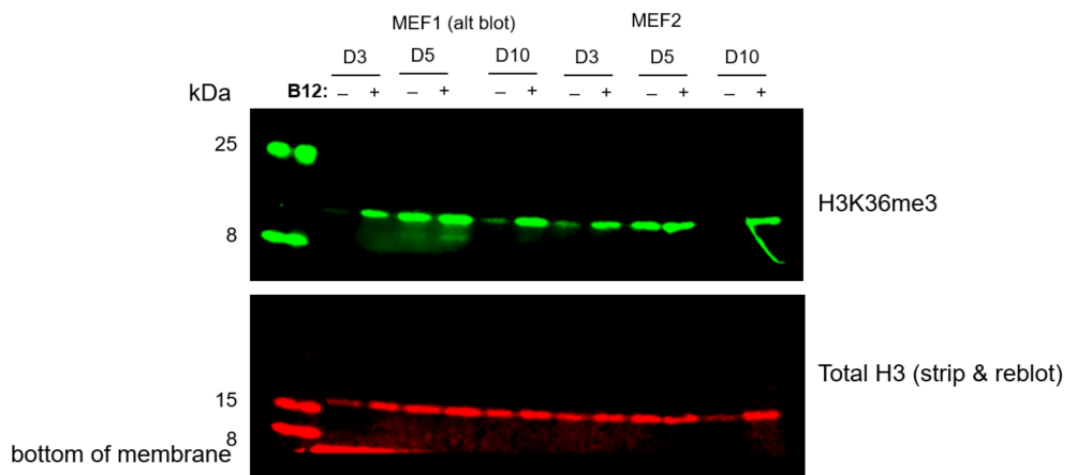

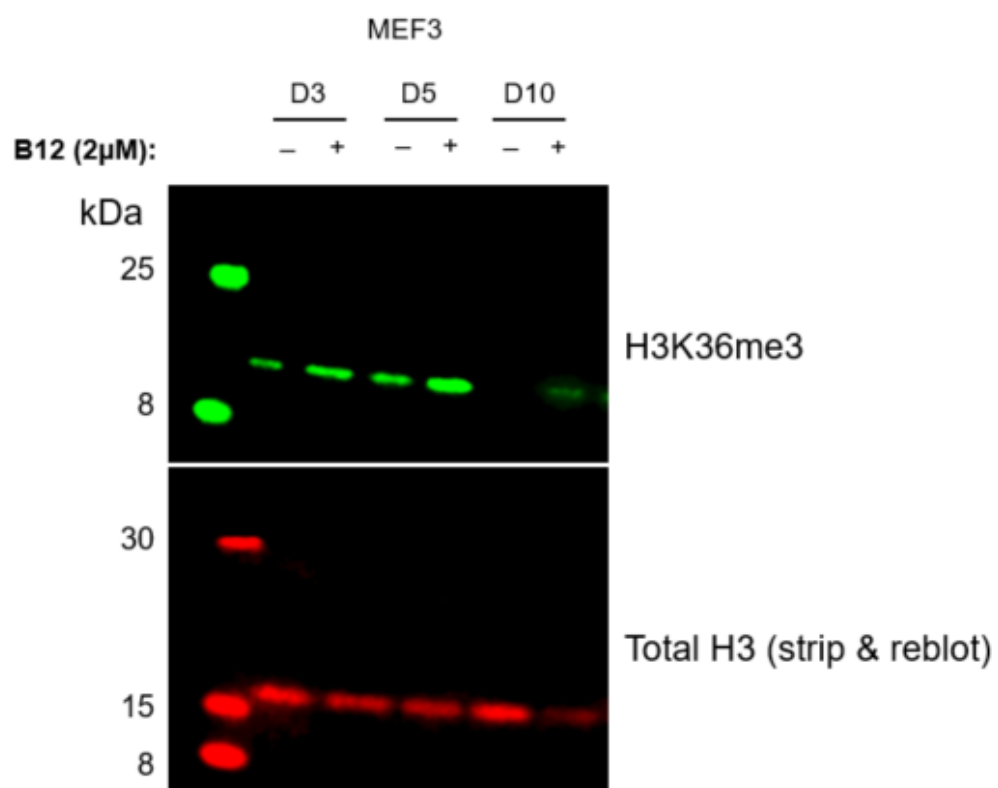

**Figure 3d Source Data:**

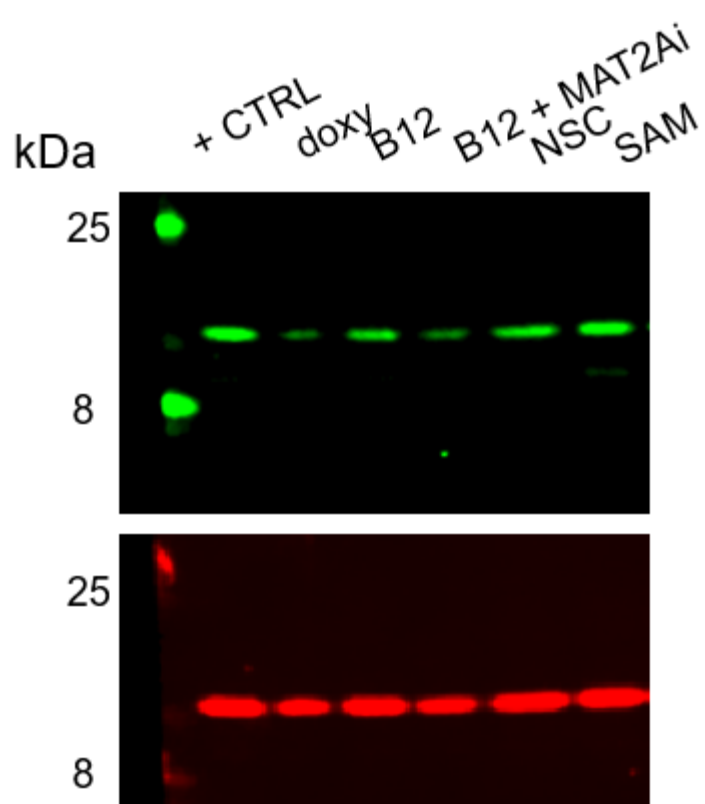

Supplement: Supplementary file 7 — Uncropped western blots. [file 42255_2023_916_MOESM7_ESM.pdf]

**Extended Data Figure 5 Source Data:**

**Extended Data Fig.5a**

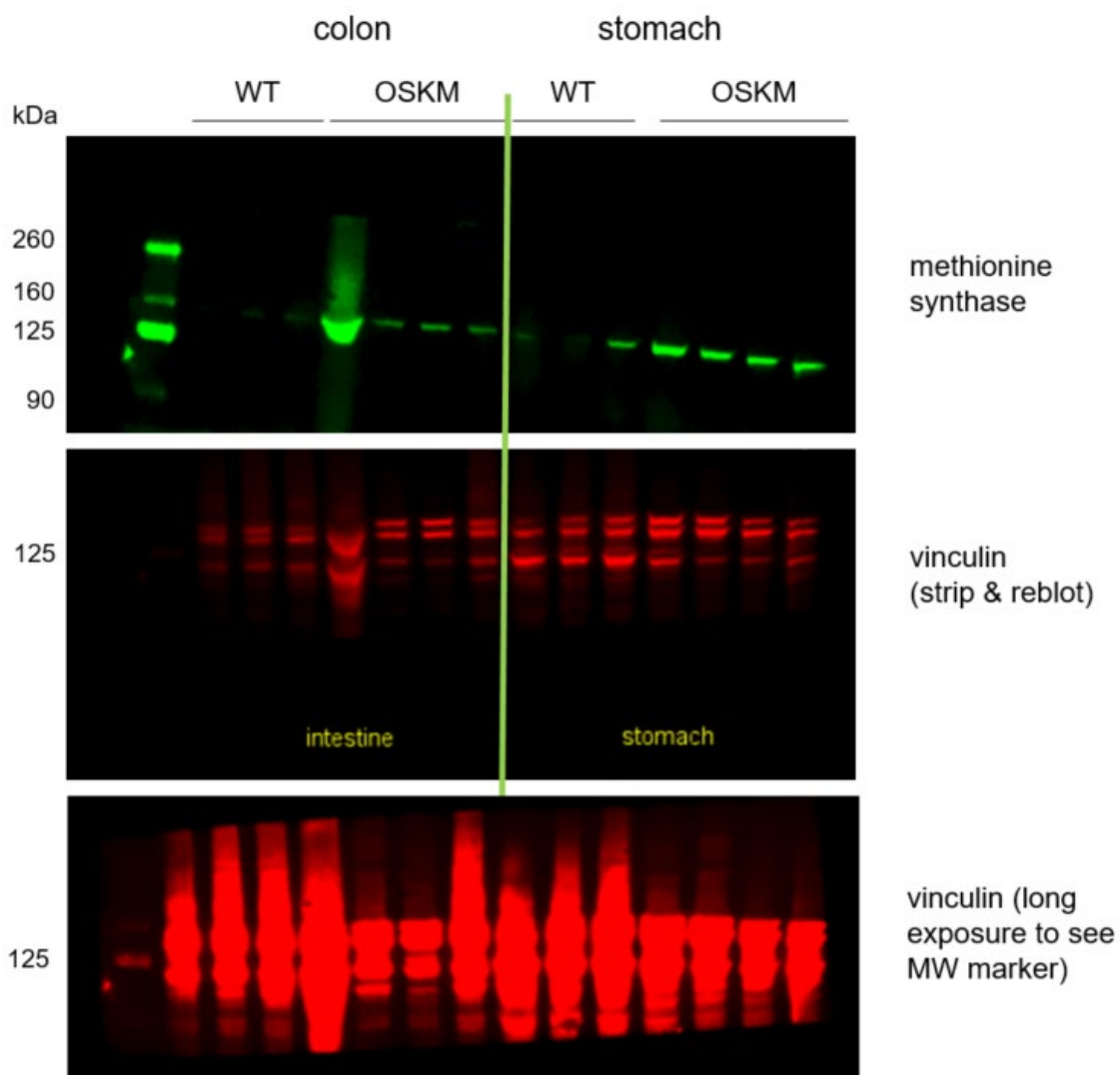

Supplement: Supplementary file 15 — Uncropped western blots. [file 42255_2023_916_MOESM15_ESM.pdf]

**Extended Data Figure 6c Source Data:**

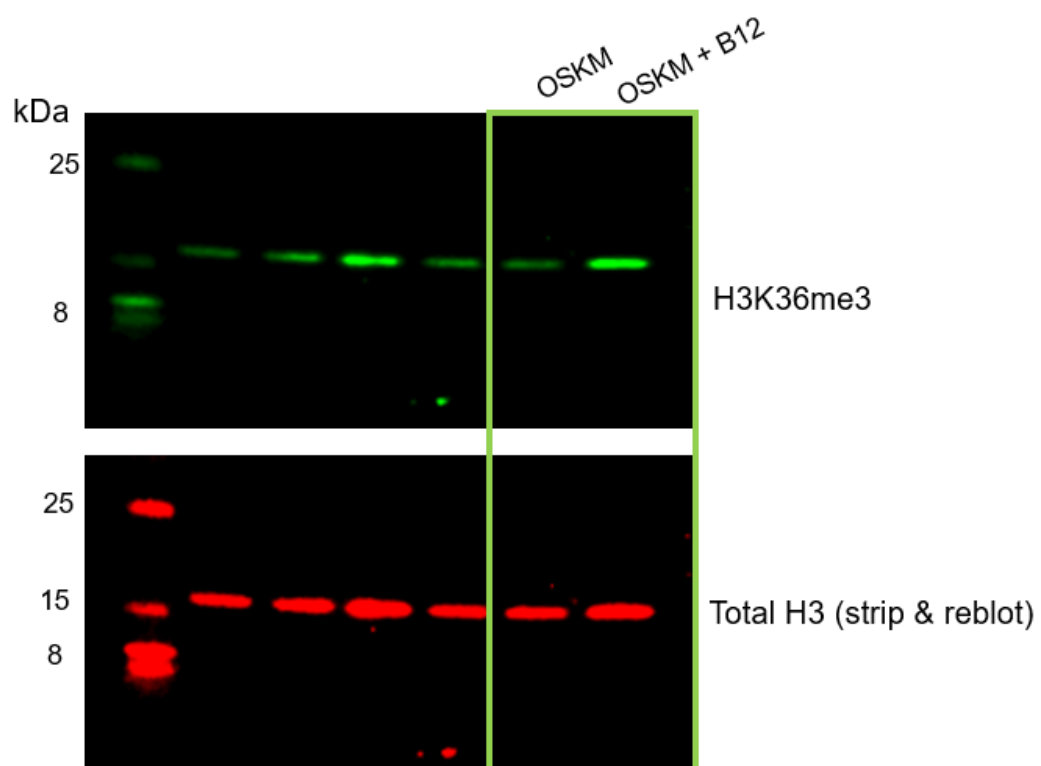

Supplement: Supplementary file 17 — Uncropped western blots. [file 42255_2023_916_MOESM17_ESM.pdf]
